# Nanoplanktonic diatoms are globally overlooked but play a role in spring blooms and carbon export

**DOI:** 10.1038/s41467-018-03376-9

**Published:** 2018-03-05

**Authors:** Karine Leblanc, Bernard Quéguiner, Frédéric Diaz, Véronique Cornet, Mónica Michel-Rodriguez, Xavier Durrieu de Madron, Chris Bowler, Shruti Malviya, Melilotus Thyssen, Gérald Grégori, Mathieu Rembauville, Olivier Grosso, Julie Poulain, Colomban de Vargas, Mireille Pujo-Pay, Pascal Conan

**Affiliations:** 1 CNRS, IRD, MIO, UM110, Université de Toulon, Aix-Marseille Université, F-13288 Marseille, France; 2 CNRS UMR 5110, Centre d’Etude et de Formation sur les Environnements Méditerranéens, Université de Perpignan Via Domitia, F-66860 Perpignan, France; 3 Institut de Biologie de l’Ecole Normale Supérieure (IBENS), Ecole Normale Supérieure, CNRS, INSERM, PSL Université Paris, F-75005 Paris, France; 4 Simons Centre for the Study of Living Machines, National Centre for Biological Sciences, UAS-GKVK Campus, Tata Institute of Fundamental Research, Bellary Road, Bangalore, 560065 India; 5 UPMC Univ Paris 06, CNRS, UMR7621, Laboratoire d’Océanographie Microbienne, Observatoire Océanologique, Sorbonne Universités, F-66650 Banyuls-sur-Mer, France; 6 CEA- Institut de Biologie François Jacob, Genoscope, 2 rue Gaston Crémieux, 91057 Evry, France; 7 CNRS, UMR 7144, Station Biologique de Roscoff, Place Georges Teissier, 29680 Roscoff, France

## Abstract

Diatoms are one of the major primary producers in the ocean, responsible annually for ~20% of photosynthetically fixed CO_2_ on Earth. In oceanic models, they are typically represented as large (>20 µm) microphytoplankton. However, many diatoms belong to the nanophytoplankton (2–20 µm) and a few species even overlap with the picoplanktonic size-class (<2 µm). Due to their minute size and difficulty of detection they are poorly characterized. Here we describe a massive spring bloom of the smallest known diatom (*Minidiscus*) in the northwestern Mediterranean Sea. Analysis of *Tara* Oceans data, together with literature review, reveal a general oversight of the significance of these small diatoms at the global scale. We further evidence that they can reach the seafloor at high sinking rates, implying the need to revise our classical binary vision of pico- and nanoplanktonic cells fueling the microbial loop, while only microphytoplankton sustain secondary trophic levels and carbon export.

## Abstract (web summary)

Diatoms are major oceanic primary producers, but some species belonging to the nano- and even picoplankton size are poorly characterized. Here the authors describe a massive spring bloom of the smallest known diatom in the Mediterranean Sea and reveal their general oversight at the global scale.

## Introduction

The widely accepted characteristics of spring blooms in marine systems are that highly turbulent front regions and eutrophic areas generally result in the proliferation of diatoms along an ecological succession sequence, which then favors coccolithophores and finally dinoflagellates as stratification and oligotrophy increase^1^. Another textbook view of the functioning of these high primary productivity events is that they contribute disproportionately to the export of carbon to the ocean interior. This concept is explicit in Legendre and Lefèvre’s bifurcation model^2^, which states that large microalgal blooms often result in increased sinking of phytoplanktonic cells or fecal pellet production, while smaller organisms are preferentially shifted toward the microbial loop, thereby reducing the fraction available for export^2^. Phytoplankton communities are typically characterized using bulk measurements, satellite data and model outputs that do not yet allow a fine scale understanding of specific floristic successions. Since the nature of the organisms composing the bloom events has dramatic effects on both higher trophic levels and biogeochemical export fluxes, it is crucial to refine our understanding of their succession determinism.

For eutrophic regions, a three-stage typical spring bloom diatom succession was proposed by Margalef^1^ and modified by Guillard and Kilham^3^. It involves a first sequence after upwelling or strong mixing that is dominated by fast-growing small species (>10 µm) belonging to the *Thalassiosira*, *Chaetoceros*, or *Skeletonema* genera, followed next by the appearance of a larger number of medium-sized *Chaetoceros* species, often forming long chains. Last, as nutrients are consumed, species more adapted to oligotrophic environments then thrive, such as large *Rhizosolenia* and *Hemiaulus* species, often associated with nitrogen-fixing cyanobacteria. The rate of succession can then be modulated by loss rates of diatoms from the euphotic zone, through diffusion, sinking, and grazing^3^. At the global scale, centric diatoms such as *Rhizosolenia*, *Chaetoceros*, and *Thalassiosira* represent a little under 50% of total diatom biomass^4^, tending to confirm this general diatom succession. However, deviations from Margalef’s typical diatom bloom scenario^1^, with very-small (<5 µm) diatom species developing into quasi-monospecific blooms have been reported on occasion, mostly in mid- and high-latitude well-mixed environments^5–10^. These phenomena are depicted as anomalous, and as a consequence, the large-scale distribution and significance of these minute diatom species are still not widely recognized.

Here, we present data relative to the 2013 spring bloom in the Northwestern Mediterranean Sea, evidencing the massive development of the small diatom genus *Minidiscus*, which overlaps both the pico- and nano-size-fractions with diameters ranging from <2 to 5 µm. We show that this tiny diatom accumulated in extremely high numbers (10^6^cells L^−1^) over a deep convection area following a particularly intense winter mixing event which extended down to the sea bottom (2400 m)^11^. Thanks to a trait-based modeling approach, we attempt to determine which key biological factors favor the proliferation of such nano-sized diatom blooms and propose that top-down control mechanisms (selective grazing, viral or bacterial lysis) may exert a specific control on the development of larger cells, opening an ecological niche for small diatoms to succeed. We then extend our results to a global scale, using metabarcoding data from the *Tara* Oceans survey^12^, showing that *Minidiscus* ranked in the top 20 most abundant diatom genera, although it has rarely been described in phytoplankton process studies. We propose, as hinted by several other authors, but still not integrated into the classical view, that nanoplanktonic diatom blooms may be more frequent than currently appreciated in open ocean and coastal areas perturbed by turbulence following vernal mixing or frontal stirring, but that their tiny dimensions and dynamics have prevented both adequate sampling and observation until now. Last, we look for evidence of the potential impact of these diatoms in carbon export, using our Mediterranean case study data from *Tara* Oceans and from literature reviews, and question whether we should revise the classical view that lower-end nanoplankton-sized cells are entirely recycled through the microbial loop and do not contribute in any significant way to carbon export to the deep ocean, as was already suggested for picophytoplankton^13^. We thus highlight these tiny, elusive diatom species as being of potential global significance in productive environments and for carbon export.

## Results

### Spring bloom of nano-sized diatoms in the Mediterranean Sea

**Fig. 1:**
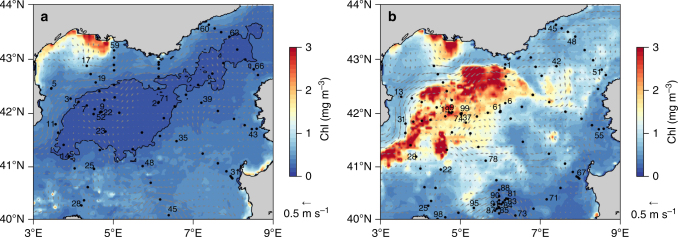
Surface chlorophyll and geostrophic circulation during DeWeX in the North Western Mediterranean Sea. Images are averages of eight days composites for both satellite-derived chlorophyll a and geostrophic velocities (in m s^−1^) over time periods corresponding to the DeWeX cruises (**a**) leg 1 (03–21 February 2013) and (**b**) leg 2 (05–24 April 2013) with a resolution of 4 km. Black dots correspond to CTD (conductivity temperature depth) casts. The black contour line shows the impoverished area with Chlorophyll *a* (Chl*a*) during leg 1 (<0.2 mg Chl*a* m^−3^) and indicates the location of the winter deep convection area, which extended until the seafloor (2400 m) in 2013. Station numbers are labeled where discrete phytoplankton samples were collected for diatom cell counts

The Gulf of Lions in the northwestern Mediterranean Sea is the hotspot for the recurrent deep-water formation during winter, due to significant autumnal and winter heat losses caused by strong Northern winds, and the presence of a cyclonic gyre enclosing dense water in the middle of the basin^14^. An intense convection event occurred during the Deep Water Experiment (DeWeX) in 2013, leading to mixing of the water column from the surface to the seafloor at 2400 m depth^15^. This massive overturn induced fertilization of the surface waters with upwelled nutrients and triggered a large phytoplankton spring bloom (Fig. 1a, b). Such an annual event is well known and recurrently observed, both from space^16,17^ and from field campaigns^18^. However, the nature of the phytoplankton bloom in this area is poorly characterized, even though it has been estimated to contribute ~15% of the primary production in the Mediterranean Sea^16^.

**Fig. 2:**
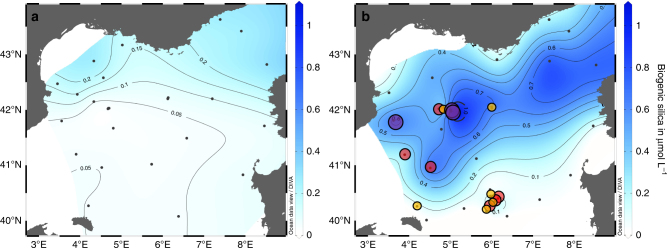
Biogenic silica distribution during DeWeX and *Minidiscus* distribution and abundance. Surface distribution of biogenic silica in blue (Ocean Data View interpolation) during (**a**) leg 1 (03–21 February 2013) and (**b**) leg 2 (05–24 April 2013) of the DeWeX cruise. A spring bloom of *Minidiscus* (2–5 µm) was reported during leg 2, at 17 out the 32 stations sampled, principally over the deep winter convection area and in an anticyclonic eddy South of the study area. Small yellow circles correspond to abundances comprised between 5000 and 100,000 cells L^−1^, medium red circles to between 100,000 and 1,000,000 cells L^−1^ and large purple circles to between 1 and 6 million cells L^−1^. The northeastern area where BSi also accumulates during leg 2 is due to larger microplanktonic sized diatoms such as *Guinardia delicatula*

In such a turbulent eutrophic environment, small- to medium-sized chain-forming diatom genera are usually expected to proliferate first upon alleviation of light limitation, followed by larger species as nutrients are consumed. However, phytoplankton determination during the DeWeX spring bloom revealed a different diatom community, with a massive accumulation dominated by at least two species of the smallest known centric diatom genus *Minidiscus* (*M. trioculatus* and *M. comicus*). They belong to the very-small end of the nanophytoplankton size-fraction (~2–5 µm diameter in our observations) even though they are reported to extend to the pico-size-fraction with known minimum diameters for *M. trioculatus* and *M. comicus* of 1.5 and 1.9 µm, respectively^19^. These species reached very-high abundances (4–6 million cells L^−1^ at several stations in April—Leg 2) and were the dominant diatoms over a large region (17 out of 32 stations) of the study area (Fig. 2a, b and Supplementary Figs. 1 and 2). Their abundance peaked mainly in the center of the convection region at around 42°N, 5°E and in an anticyclonic eddy south of the study region around 40.5°N, 6°E, where abundances ranged from 5000 to 425,000 cells L^−1^. Meanwhile, microphytoplanktonic diatoms (>20 µm) were only observed closer to the Gulf of Lions plateau in winter and in the northeastern region between the coast of France and Corsica in spring, but never exceeded 17,000 cells L^−1^, a low value for a spring bloom. *Minidiscus* was not observed at any site during February (leg 1), which suggests an earliest bloom initiation in March. Increased biogenic silica (BSi) in the areas where *Minidiscus* was absent was associated with typical larger diatoms such as *Pseudo-nitzschia* spp., *Leptocylindrus* spp., *Cylindrotheca* spp., and other large *Thalassiosira* spp. during winter and mainly to *Guinardia delicatula* and *Chaetoceros* spp. during spring (Supplementary Figs. 1 and 2). At a few stations during April (leg 2), the small pennate *Nitzschia bicapitata* (~10 µm) was also observed in elevated concentrations in association with *Minidiscus*, but the abundances of the latter were always at least 1 or 2 orders of magnitude higher. BSi concentrations associated with the densest *Minidiscus* bloom area were elevated for the Mediterranean Sea (1 µmol L^−1^) and particulate Si:C ratios (0.07–0.10) were close to Brzezinski’s values for small diatoms^20^. An estimation of the growth rates required to produce such an accumulation yields a minimum approximate net rate of 0.3 d^−1^ (for an average of 805,000 cells L^−1^) between early March and mid-April starting from a seed population of 10–100 cells L^−1^. This is higher than the rate of 0.13 d^−1^ measured in situ during a more moderate *Minidiscus* spring bloom observed in the Norwegian basin in 2012^10^, which could be explained by the lower temperature (~6–7 °C) compared to our study area (~13 °C)^21^. However, if the *Minidiscus* bloom grew over a shorter period of time, for instance over a week, growth rates would have been much higher and closer to 2 d^−1^. The unexpected very-small size structure of this phytoplankton community was confirmed by an automated flow cytometer installed on an inflow of surface water pumped continuously at 3 m depth. It evidenced that the massive spring bloom in April was almost entirely dominated by nanophytoplankton (Supplementary Fig. 3). Observations by optical microscopy revealed that the *Minidiscus* bloom co-existed with even larger numbers of undetermined nanoflagellates and cryptophytes, all smaller than 20 µm, while abundances of larger cells were about three orders of magnitude lower at most sites (Supplementary Fig. 3).

A further unusual feature was the overall dominance of diatoms by *Minidiscus* during this bloom. At all sites where *Minidiscus* was observed, it represented on average 92% of total diatom abundance (Table 1). Notwithstanding, conclusions drawn on abundance are difficult to transpose to biogeochemical relevance, in particular at both ends of the size spectrum, where abundance and biomass are no longer closely related. To assess whether this numerically abundant bloom was an important contributor to total phytoplankton biomass, we converted abundance data to carbon (C) content, using an average biovolume of 18 µm^3^ and subsequent Si and C content estimate of ~0.8 pmol C cell^−1^ and 0.08 pmol Si cell^−1^, consistently with the data given in Brzezinski (1985)^20^ for another similar sized species (24 µm^3^), but about 3-fold higher than the quotas predicted using standard allometric approaches^22,23^. *Minidiscus* biomass was then compared to measured particulate organic carbon (POC) and BSi concentrations in the same samples. Although *Minidiscus* was not the dominant contributor to C biomass given its small size, the high abundances of other nanoplankton groups and the variable content of dead material in POC, it still reached between 18 and 26% of total POC at stations 74 and 99 (Table 1), located close to the center of the convection area (42°N, 5°E). At station 74, *Minidiscus* constituted the bulk of in situ BSi stocks (99.4%) on one occasion on April 19^th^, whereas its relative contribution decreased to 36% by the end of April at the same location (Table 1).

**Table 1:** *Minidiscus* spp. cell counts and relative contribution to abundance and biomass

Date (DEWEX Leg2)	Stations number	Latitude °North	Longitude °East	*Minidiscus trioculatus* (cells L^−1^)	% *Minidiscus* contribution to total diatom abundance	% *Minidiscus trioculatus* contribution to POC	% *Minidiscus trioculatus* contribution to BSi
05/04/13	1	42.91	6.12	0	0	—	0
06/04/13	6	42.05	6.00	15 250	52.1	—	0.2
07/04/13	9	42.03	4.80	76 453	81.9	1.2	2.1
07/04/13	13	42.31	3.51	0	0	—	0
08/04/13	19	42.02	4.72	557 645	98.6	10.8	9.5
09/04/13	22	40.95	4.51	454 725	95.1	3.8	6.0
10/04/13	25	40.26	4.19	2 711	96.4	0.0	0.5
10/04/13	28	41.19	3.89	162 228	84.5	—	5.4
11/04/13	31	41.81	3.62	1 025 696	98.4	6.4	14.6
11/04/13	37	42.00	5.00	268 172	98.8	1.6	3.7
12/04/13	42	42.88	7.40	0	0	—	0
13/04/13	45	43.63	7.39	0	0	0	0
13/04/13	48	43.42	7.87	0	0	0	0
14/04/13	51	42.81	8.53	0	0	0	0
15/04/13	55	41.71	8.46	0	0	—	0
16/04/13	61	42.03	6.00	1 300	100	0	0
17/04/13	67	40.82	7.91	0	0	—	0
18/04/13	71	40.38	7.16	0	0	0	0
18/04/13	73	40.08	6.37	0	0	0	0
19/04/13	74	41.98	5.02	5 819 040	99.0	18.3	99.4
19/04/13	78	41.12	5.63	0	0	0	0
21/04/13	81	40.40	6.15	174 368	98.0	1.2	19.6
22/04/13	83	40.33	6.06	425 316	99.1	3.6	18.9
22/04/13	84	40.29	6.02	91 554	84.3	0.6	5.1
22/04/13	85	40.24	5.95	274 234	98.2	2.3	13.6
22/04/13	87	40.17	5.87	0	0	0	0
22/04/13	88	40.56	5.96	5 746	98.8	0.1	4.7
22/04/13	89	40.44	5.97	0	0	0	0
23/04/13	91	40.30	5.97	33 212	87.5	0.3	3.6
23/04/13	95	40.22	5.33	0	0	0	0
24/04/13	98	40.01	4.42	0	0	0	0
24/04/13	99	41.98	5.02	4 307 310	99.4	26.4	36

Contribution to total diatom abundance, particulate organic carbon (POC) and biogenic silica (BSi) stocks during leg 2 of DeWeX

This locally significant contribution is surprising given that the genus *Minidiscus* has never been documented to form such intense blooms in the Mediterranean Sea. *Minidiscus* is in fact absent from many taxonomic books for this basin, and appeared for the first time in an inventory of the Catalan Sea only in 1992^24^. *M. comicus* was only recently observed in the same region of the northwestern Mediterranean during spring^25^, and in nearby regions of the Gulf of Naples^26^, while *M. trioculatus* was considered to be rare. *Minidiscus* was observed for the first time in April 2012 at a coastal bi-monthly time-series site located on the Gulf of Lions coastal shelf in the Bay of Marseille (SOMLIT), at very low baseline abundances. Higher abundances were reported in May and July 2013 following our study (B. Beker, personal communication). The only other report of such massive nano-sized diatom bloom in the Mediterranean Sea was that of the small centric diatom *Thalassiosira partheneia* (<8 µm) in the Almeria-Oran front^6^, where it accumulated in a thin layer at depth up to ~10 million cells L^−1^ with a similar calculated net growth rate of 0.2 d^−1^.

**Fig. 3:**
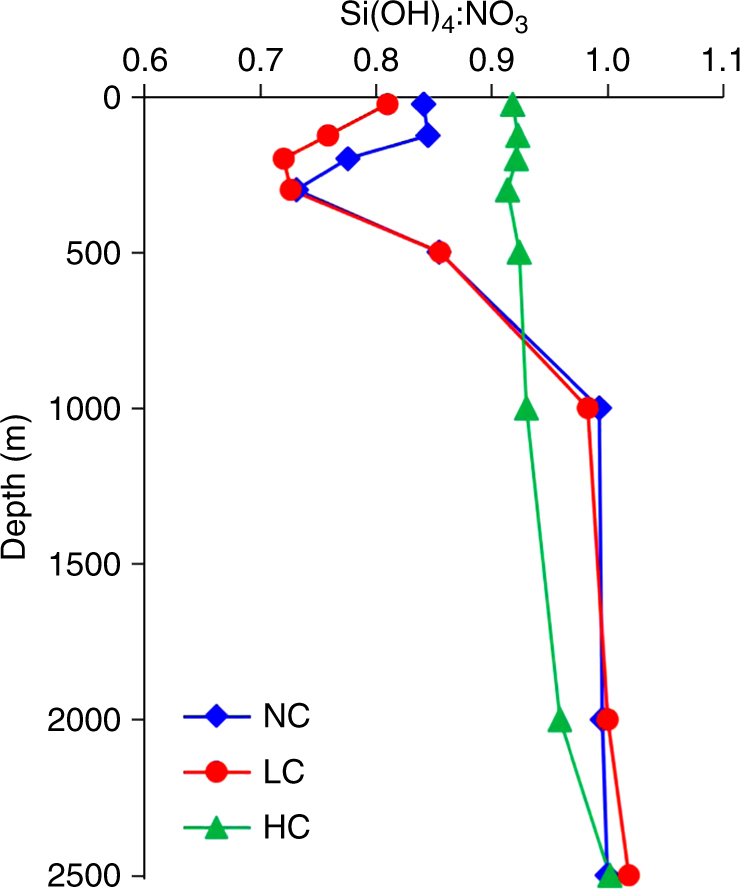
Vertical Si-N nutrient ratio profiles in different regions of the DeWeX cruise. H_4_SiO:NO_3_^−^ (mol:mol) ratio profiles in three station clusters (NC: No Convection, LC: Low Convection, HC: High Convection) based on hierarchical clustering of the depth extent of the winter mixing^11^

The nutrient concentrations at stations sampled during winter revealed that the amount of nutrients upwelled to the euphotic layer was dependent on the area covered by the convection event, while nutrient stoichiometry was dependent on the vertical extent of the convection depth^11^. From a hierarchical ascendant classification analysis of stations sampled during winter (DeWeX cruise leg1), we found that highly convective regions were characterized in the surface layer (0–50 m) by high H_4_SiO_4_ and NO_3_^−^ concentrations (7.7 µM and 8.4 µM, respectively), while weakly convective regions showed much lower nutrient content (2.1 µM Si and 2.7 µM N, respectively)^11^. In parallel, the Si:N nutrient ratio prior to the spring bloom in the surface layer was 15% higher in the case of high convection compared to low convection or no mixing at all (Fig. 3). High convection events mixing deep water masses with the surface layer thus seem necessary to supply enough Si to the surface relative to N and P to sustain a high bloom situation dominated by diatoms^27^. The dominance of *Minidiscus* during DeWeX could thus partly be attributed to their higher efficiency with respect to larger diatoms for taking up high silicic acid concentrations, which were preferentially upwelled compared to nitrate^28^. In the absence of physiological rate measurements during the cruise, we rely on the following modeling exercise (not coupled to physical forcings) to test which bottom-up or top-down processes could best explain the preferential development of nano-sized diatoms over larger species.

**Fig. 4:**
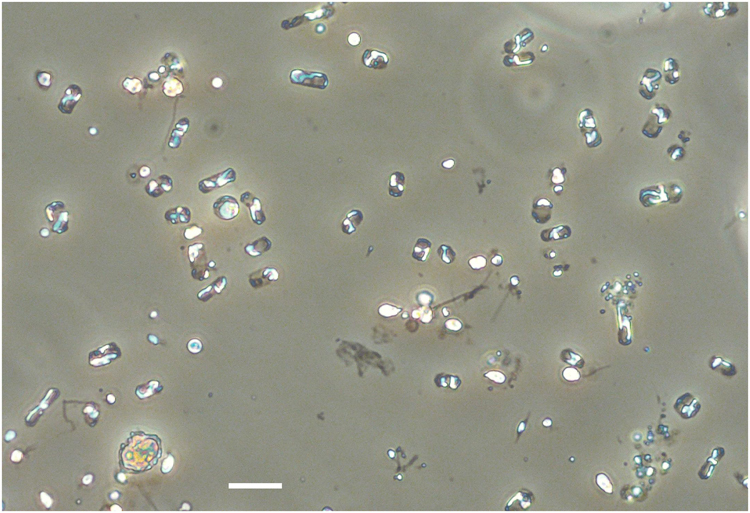
*Minidiscus* spp. in light microscopy. The dominating nano-sized *Minidiscus* centric diatoms (2–5 µm diameter) seen in light microscopy at station 74 (5.8 million cells L^−1^) showing the impossibility of identifying it with the latter technique, while it remains possible to count it and compare with parallel SEM identification. The yellow Lugol stain background was removed using the camera’s autowhite function. The scale bar is 10 µm

**Fig. 5:**
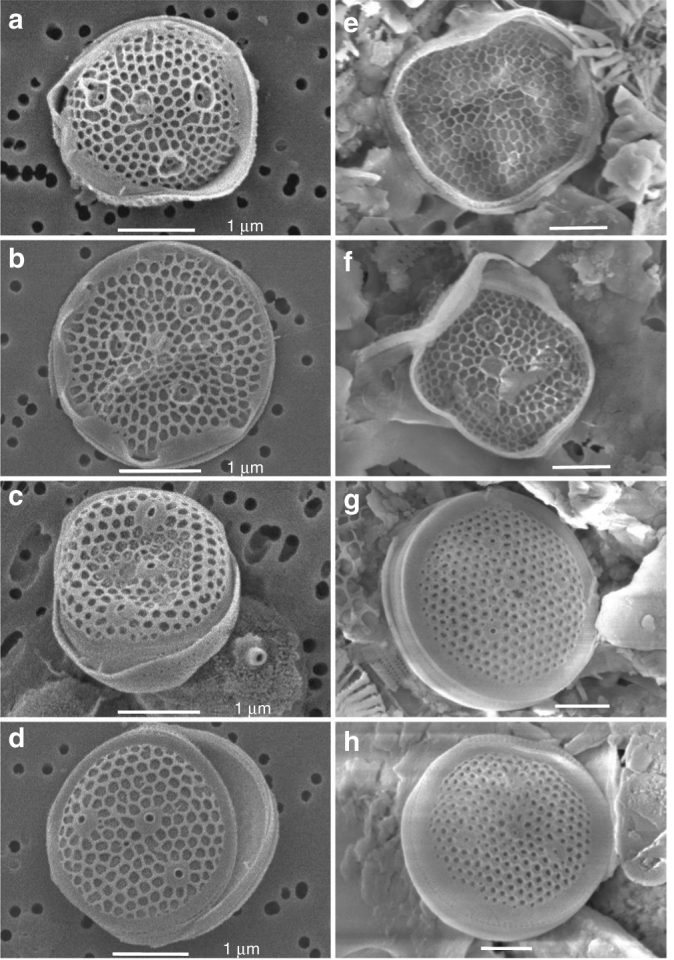
*Minidiscus comicus* and *Minidiscus trioculatus* in surface water and sediment trap samples during spring 2013. (**a**, **b**) *M. comicus*, (**c**, **d**) *M. trioculatus* observed in scanning electron microscopy (SEM) at station 99, located at 42°N, 5°E on 24^th^ April 2013 at the surface. (**e**, **f**) *M. comicu*s, (**g**, **h**) *M. trioculatus* observed in SEM in three deep sediment traps samples (2400 m) at 42°N, 4.5°E covering 30^th^ March to 22^nd^ May 2013. All scale bars are 1 µm

Biogeochemical models are often based on two main numerical representations of biota, the plankton functional type (PFT) models that are now widely used^29^ and the trait-based models grounded on the Reynolds’ C-S-R classification^30^ built on Margalef’s mandala^1^. On one hand, PFT-based models only depict diatoms as a single large phytoplankton box connected to the Si cycle and are, therefore, inherently unable to reproduce different life strategies and traits within diatom community. On the other hand, the C-S-R models can be applied in the present context as an adapted tool to represent a spectrum of different strategies of development among diatoms. Each of these strategists is characterized by a specific ecological niche, defined by a combination of nutrient and light variables, which are the major factors impacting diatom survival strategies^31^. We used a simple C-S-R trait-based model representing four different types of strategists to attempt to understand the reasons underlying the occurrence of the massive bloom of *Minidiscus* evidenced during DeWeX in 2013. Among the four considered strategists, two diatom types were differentiated. Owing to its morphological and physiological traits, *Minidiscus* was considered in the present model as a colonist genus (i.e., a C-strategist). This group is characterized by a high growth rate, a small size and a round shape (Figs. 4 and 5). The other diatom type, characterized by higher growth rates, larger sizes and elongated shapes, such as *Chaetoceros*, groups light-stress tolerant ruderal species (i.e., R-strategists). Using conditions based on the measured light and nutrient levels prevailing prior to the 2013 spring bloom^27^, the results of our trait-based model show a large and rapid dominance of R-strategist diatoms (Supplementary Fig. 4 and 5), which is not in line with the observations of the *Minidiscus* bloom. Given the nature of our model, this result suggests that some processes occurring in the field other than bottom-up factors may prevent the bloom of R-strategists, such as for instance a sharp increase in their mortality rate, which in turn may favor the emergence of C-strategists (i.e.*, Minidiscus*) by escaping predation. When higher mortality rates are applied to the R-strategists, the model correctly simulates a rapid dominance of C-strategists. Although the numerical model is unable to determine which exact process is driving the prevalence of C-strategists over R-strategists in the field, it nonetheless offers a plausible hypothesis that a selective top-down control on larger diatoms is necessary given each strategist’s defined niche and environmental parameters matching the DeWeX dataset. In situ, viral lysis, attacks by various pathogens^32^, differential sensitivity to mixing, preferential grazing as well as larger differences in the traits between *Minidiscus* and others diatom genera are some of the known processes that may exert control on abundance of large diatoms. A transient lower vulnerability or accessibility of *Minidiscus* to grazers may also be an indirect mechanism involving its success during DeWeX. It is known that *Minidiscus* can produce some protruding organic threads depending on turbulence conditions^33^ and that these threads would increase its vulnerability to copepod grazing^34^. Unfortunately, these delicate threads are only preserved up to three months in glutaraldehyde and dissolve in less than 15 days in Lugol^34^, which made us unable to confirm their presence. The upscaling of mortality rates for large diatoms in the model is further supported by the observations made on the zooplankton community during DeWeX^35^. The deep convection zone where *Minidiscus* prevailed was characterized by high abundances of large herbivorous genera (such as *Centropages, Calanus)* while smaller grazers (e.g., *Microsetella, Oncae*) were much less abundant. Trophic pathways of phytoplankton community through the zooplankton food-web determined using stable isotopes during DeWeX, also revealed that the nanoplankton size-class made the largest contribution to zooplankton biomass during winter, while the high convective area was characterized by the largest contribution of microplankton to zooplankton biomass during spring^36^. Hence, an ecological framework for nanoplanktonic diatom blooms can be postulated from this simple trait-based model and supported by observation. Small diatoms would be likely dominant during the early bloom phase in both low and high convective areas but in the latter case only a strong top-down control on R-strategists (by viral/bacterial pathogenesis, parasites or grazing) would allow nano-sized species, such as *Minidiscus* to reach the observed high bloom intensities.

### The case for large-scale oversight of nano-diatoms

**Fig. 6:**
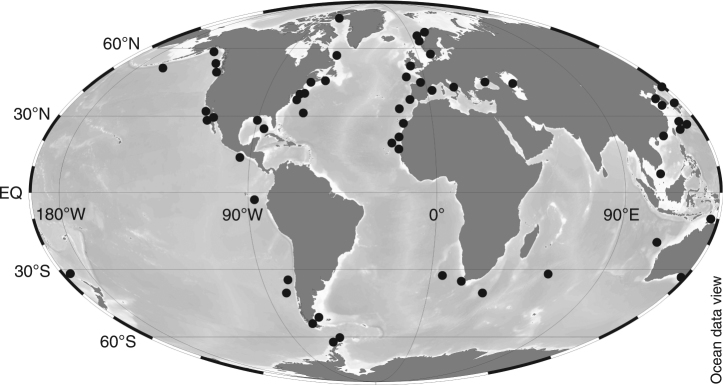
Biogeographical distribution of *Minidiscus* across ocean basins. Information regarding the biogeographical distribution of *Minidiscus* spp. was derived from a literature review (from references ^19,37–39,80–95^)

The genus *Minidiscus*, composed of only a few reported species, was initially described in 1973 in the Norwegian Sea^37^. It is considered a cosmopolitan genus^38^, having been observed in all oceanic basins, including northern and southern polar environments (Fig. 6). However, this genus goes frequently unnoticed because it is easily overlooked in conventional microscopy, and is often misidentified and/or systematically under-sampled by net hauls due to inappropriate mesh sizes. When discrete water sampling is adequate, pico- and nano-sized diatoms may be enumerated using light microscopy but can only be determined to the genus and species levels using scanning electron microscopy (SEM) (Fig. 5), a difficulty that has already been emphasized for *Minidiscus*^9,38–40^. Only a few other studies have documented significant bloom events of nano-sized diatoms. Blooms of *Minidiscus* spp. have for instance been reported during a 14-year survey in Monterey Bay, a coastal region characterized by strong upwelling events, an ecological situation similar to that of the DeWeX study^9^, as well as in other eutrophic areas of the Subarctic Pacific^5^, Norwegian Basin^10^, and Antarctic Peninsula^39,41^. In the Mediterranean Sea, a massive bloom (~10 million cells L^−1^) of the nano-sized diatom *Thalassiosira partheneia* (<8 µm) was reported near the Gibraltar Strait^6^. Large spring blooms of the tiny pennate diatom *Nanoneis hasleae* (2 × 5 µm) have also been reported on a few occasions in the North Atlantic and may be similarly overlooked on a global scale^42,43^. Nanoplanktonic biomineralizing algae, including diatoms, Parmales (siliceous plate-bearing phytoplankton <5 µm) and coccolithophores were also found to have been largely underestimated in the Southern Ocean^44^.

It, therefore, seems possible that *Minidiscus* together with other nanoplanktonic sized diatoms, may be responsible for occasional massive blooms, which may go undetected due to either collection or identification biases. This is further exemplified in the global diatom database compiled during the MAREDAT project^4^, which presented close to 10,000 unique georeferenced locations and 607 reported diatom species since the 1930 s, in which nano-sized genera such as *Minidiscus, Minutocellus, Cyclotella, Lennoxia*,or *Nanofrustulum* are completely absent. This absence suggests that, if adequately sampled at all, they were either confused with similar looking species such as small *Thalassiosira* species, counted as undetermined species, or simply not even recognized as diatoms.

### *Tara* Oceans metabarcoding data

**Fig. 7:**
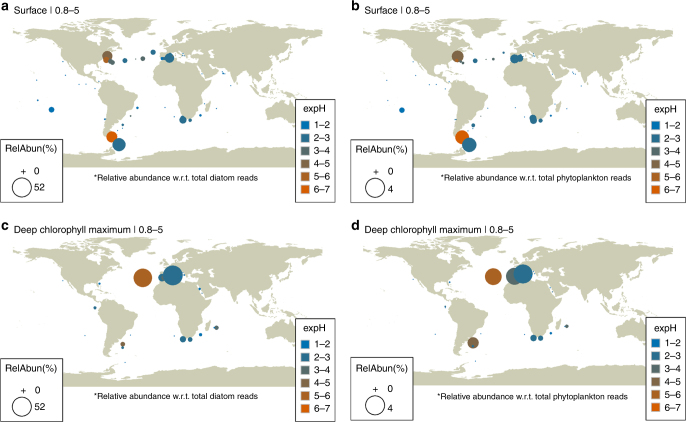
Biogeographical distributions of *Minidiscus* from metabarcoding data. Biogeographical distributions at the surface (SRF) and deep chlorophyll maximum (DCM) depths of genus abundance and diversity of *Minidiscus* as relative abundance of total diatom reads (**a**–**c**) and as relative abundance of total phytoplankton reads (**b**–**d**) in the 0.8 to 5 µm size-fractions collected during the *Tara* Oceans expedition (2009–2013)^77^. The variation in diversity for each genus is indicated as the exponentiated Shannon Diversity Index (expH) and the color represents the number of unique ribotypes (blue = low richness; orange = high richness). Bubble symbols are scaled to indicate the relative percent reads of each genus with respect to total diatoms or total photosynthetic reads in the sample

The advent of high-throughput sequencing now allows unprecedented access to pico- and nano-plankton, as they can be detected even at low levels in filtered samples, thus circumventing both sampling and observational biases. Analysis of the metabarcoding-based descriptions of eukaryotic plankton by *Tara* Oceans^45,46^ indeed reveals the predominance of nano-sized diatoms such as *Minidiscus* (Fig. 7) and *Minutocellus* (Supplementary Fig. 6). Their ubiquitous biogeographical distribution confirms and significantly extends previous observations, in particular regarding open-ocean systems, as previous biogeography described in the literature evidenced *Minidiscus* mostly in coastal environments (Fig. 6). Significantly, *Minidiscus* is in the top 20 most abundant diatom genera (Supplementary Fig. 7) even though it has never been described as a major bloom-forming species^46^. Supporting our previous observations made during DeWeX, the distributions from the *Tara* Oceans data show that the relative abundance of *Minidiscus* was the highest in the Mediterranean Sea, followed by the Southern Ocean and the North Atlantic Ocean (Supplementary Fig. 8a). Furthermore, the data reveal that *Minidiscus* is not only abundant at the surface but also in samples taken from deep chlorophyll maxima (DCM) contributing to what is known as the shade flora which benefits from the best ratio between sufficient light and upward limiting nutrient fluxes (Supplementary Fig. 8b). Size-class fractionations further confirmed that *Minidiscus* and *Minutocellus* were mostly represented in the smaller size-fractions, but also occurred in larger size classes probably due to cell aggregation (Supplementary Fig. 8c). A recent study has also transferred two *Thalassosira* species to the *Minidiscus* genus after fine structure examination and molecular sequence comparisons^47^, illustrating the difficulty of correct identification in this size-class. These recent changes have been included in the present *Tara* Oceans data analysis, but do not significantly change the global scale picture. However, while sequence information is available from *M. trioculatus* cultures, this is not yet the case for *M. comicus* implying that *Tara* Oceans data underestimate *Minidiscus* abundances. Also recently, *Minutocellus* has been shown to be a symbiotic species of the foraminifera *Pararotalia calcariformata* in the Mediterranean Sea^48^, which is another factor explaining why they may be overlooked in phytoplankton samples and why they are more important in the large size-fraction of the *Tara* Oceans samples than *Minidiscus* (Supplementary Fig. 7c). These results, together with previous observations, build a strong case for a large-scale oversight of these genera, together with many other nano-sized diatom species in the marine realm.

### Contribution of nanoplanktonic diatoms to carbon export

In order to examine the impact of small-sized diatoms to carbon export to depth, we collected samples from a deep sediment trap (2400 m) moored at 42°N, 4.5°E from the end of March to the end of May 2013 during the DeWeX study, below the main area where *Minidiscus* reached ~6 million cells L^−1^. SEM observations of samples from the three sediment trap samples collected during that period revealed the presence at high abundances of both *M. trioculatus* and *M. comicus* frustules (Table 2 and Fig. 5e–h). Due to the low representativity of SEM counts (small area imaged and heterogeneous nature of the collected material), accurate *Minidiscus* flux calculations were not attempted. However, given the time-lag between surface observations and sediment trap collection of *Minidiscus*, about one month assuming a bloom initiation in early March, a rough estimate indicates that a minimum sinking rate of 80 m d^−1^ is likely, which is in the range of literature values for phytoplankton aggregates^49,50^. It was also inferred from the vertical distribution and temporal evolution of particle concentrations in the water column during the DeWeX cruises^51^ that particulate matter was primarily composed of aggregates with settling rates of ~1 mm s^−1^ (i.e., 86 m d^−1^), which supports the previous hypothesis of a rapid sinking of *Minidiscus* as phytoplanktonic aggregates. Unidentifiable clumped aggregates were observed in SEM, but could not be discriminated with certainty between fecal pellets or phyto-aggregates due to the long-term storage of these samples between sampling and observation (4 years). It was already noted using culture collections of *M. trioculatus* that single cells growing exponentially became aggregated in compact clusters during stationary phase^40^. It is further known that these species are able to grow thin chitin threads protruding from the strutted processes of the valves, a feature considered unique to Thalassiosiroids^52^ and modulated by turbulence^33^. Finally, *M. comicus* is known to form small colonies of 2–3 cells connected with extruded threads^40^. Clearly, these features could if present, increase aggregation and sinking rates significantly^33,34^.

**Table 2:** Observations of *Minidiscus* valves in the LIONCEAU sediment trap samples during DeWeX

Trap sample (42°N, 4.5°E, 2400 m)	Date of sampling	*Minidiscus* valves
LIONCEAU 9	31/03/13–15/04/13	abundant
LIONCEAU 10	15/04/13–30/04/13	abundant
LIONCEAU 11	30/04/13–23/05/13	abundant

The LIONCEAU sediment trap was located at 42°N 4.5°E at 2400 m depth at the center of the DeWeX deep vernal convection area and close to where the *Minidiscus* bloom was most elevated in surface samples. *Minidiscus* valves were searched in the sediment trap samples in SEM and were found to be abundant in all three

At least three other studies carried out at both northern and southern high latitudes have revealed high abundances and even a dominance of *Minidiscus* in sediment traps. The first study was carried out in relatively shallow sediment traps (100–200 m) during a temporal survey from 1985 to 1987 in a Vancouver Island inlet where abundant contributions of both *M. chilensis* and *M. trioculatus* were noted in spring and winter^53^. Other studies carried out near the Antarctic Peninsula reported high abundances of *M. chilensis* in surface waters^39,41^, and the annual survey conducted in 1998 in the Bransfield Strait revealed massive *M. chilensis* fluxes in 1000 m deep trap samples, representing up to 87% of total diatom flux and associated to elevated BSi flux (~150 mg Si m^−2^ d^−1^)^39^.

In support of the hypothesis of a rapid sinking of nano-diatoms, *Tara* Oceans data show that both *Minidiscus* and *Minutocellus* cells are consistently present in multiple mesopelagic (MESO) samples collected at around 700 m depth (Supplementary Fig. 8c) while, on average, diatoms represent up to 46% of photosynthetic read abundance at 700 m. Furthermore, with respect to samples collected in the photic zone, these small diatoms are present at higher proportions with respect to other diatom genera, e.g., *Minidiscus* is the eighth most abundant diatom genus in MESO samples and the 21^st^ most abundant in photic zone samples (Supplementary Fig. 7). The conclusion from these multiple observations is therefore that small nano-sized diatoms are able to contribute to deep-sea carbon injection, even though this compartment is not usually considered to fuel export.

## Discussion

*Minidiscus* was described for the first time in 1973^37^ and has traditionally been considered a rare genus, but the results reported here show on the contrary that it ranks among the most abundant diatom genera in the global ocean. The most likely explanation for this apparent discrepancy is that traditional collection and identification methods were unable to detect and taxonomically resolve such small-sized species. Flow cytometric analyses do not resolve diatom taxonomy and only detect these small diatoms as undetermined nanoplanktonic eukaryotes, while the diagnostic pigments approach^54^ usually attributes all fucoxanthin to the microphytoplankton size-class, which is obviously erroneous for the DeWeX case study. Much like the application of flow cytometry was required to detect the significance of *Prochlorococcus* in the global ocean^55^, the advent of improved genomic sequencing performed on a global scale and systematic coupling of faster SEM techniques to regular optical microscopy should help to further resolve this diatom size class in coming years. But clearly, quantitative physiological rates will be needed to improve model parameterizations.

Diatom cell sizes, as well as their Si/C cellular quotas, are key variables controlling their sinking fluxes^56,57^. In the North Atlantic in 1989–1990, a nearly two-fold reduction in carbon flux to deep sediment traps occurred during two successive years following the spring diatom bloom^7^. In 1990, the spring bloom was dominated by the tiny pennate *Nanoneis hasleae* forming a bloom as large as 50,000 km^2^ visible from space^43^, while it was dominated by a large size chain-forming *Chaetoceros* assemblage in the previous year^7^. This was one of the first studies to document a direct effect of the community structure within the same PFT on the modulation of carbon export fluxes to depth. This scenario was consistent with the classical opposition between small solitary diatoms carrying no protective spines correlated to slower sinking rates and faster C remineralization (through either bacterial lysis or grazing) in the water column compared to larger chain-forming and/or spiny cells, prone to aggregation and fast sinking. The data collected during the DeWeX spring bloom from deep sediment traps adds, however, a further aspect to this scenario, demonstrating that they are able to sink out of the surface layer in aggregated form at high sinking rates contrary to the common assertion that they are more likely to be entirely remineralized within the microbial loop in the water column. There is, therefore, a need to better constrain sedimentation rates and aggregation processes from different sizes of diatoms. This focus probably needs to be extended to other nanoeukaryotic groups, such as Parmales, another elusive siliceous scale-bearing phytoplankton of 2–6 µm diameter, whose importance has likely been underestimated^44^. Nano-diatoms and Parmales are ballasted by their mineral casings, which should lead to a different fate in the water column with respect to other non-siliceous organisms of the same size-class. The possibility for picoplankton to escape the microbial loop and fuel export was already demonstrated by sediment trap data^13^, but to date it has not been considered extensively. We further propose that the diatom group represented in models as a single PFT should be redefined to better include various life strategies of survival and growth, such as in trait-based models, and that the nanophytoplankton group also needs to be connected to the siliceous pathway.

Another outcome of the modeling study is that traits of diatoms concerning their relationships to predators need to be further explored. Size-selective predation, which can include zoosporic parasitism^32^, rather than competition for resources may be a major selective pressure for diatoms, driving their ecological properties^3^. At least two other studies reporting spring bloom abundances of *Minidiscus* sp. in the Subarctic Pacific^5^ and in the Norwegian basin^10^ displayed similar context and pointed toward the same explanation. Both studies argued that despite generous supplies of nutrients, usually favorable to the growth of large cells, the net accumulation of small-sized diatoms could be a result of selective grazing pressure on larger cells and possibly linked to a lack of seed populations from coastal environments during the initial stages of the bloom. None of these studies, including ours, were able to precisely determine the interspecific predator-prey relationships between zooplankton groups and distinct diatom species. Indeed, determining trophic links in the natural environment remain a major challenge and constitutes a bottleneck in our understanding of marine planktonic ecosystems. Understanding of accessibility, vulnerability, prey-induced biases in the predator perception, and trophic relations with organisms other than copepods is still in its infancy and needs to be scaled up. The same is true about symbiotic interactions such as the one recently discovered between *Minutocellus* and a foraminifera. The advent of improved genomic sequencing and systematic coupling of faster SEM techniques to regular optical microscopy should help to further resolve this diatom size class in coming years.

Altogether, these observations challenge the common assumption that small (defined as >10 µm) to large-sized chain-forming diatoms are the usual initial bloomers in frontal areas and turbulent environments following winter fertilization^2^. We propose that *Minidiscus* and other overlooked minute diatom taxa (<5 µm) may be occasional major contributors to spring blooms in turbulent nutrient-rich environments of various coastal and offshore oceanic regions and that, upon aggregation, they may also contribute significantly to carbon export.

## Methods

### Satellite products

Satellite-derived surface chlorophyll *a* concentration was accessed at http://oceancolor.gsfc.nasa.gov (MODIS Aqua, 4 km, 8-days composite, level 3 product). Altimetry-derived geostrophic velocities (AVISO MADT, 1/4°, daily product) were extracted at http://www.aviso.altimetry.fr/. Both satellite-derived chlorophyll *a* and geostrophic velocities were time-averaged over periods corresponding to the DeWeX cruises leg 1 (03–21 February 2013) and leg 2 (05–24 April 2013) to produce the maps in Fig. 1.

### DeWeX cell counts and taxonomical determination

Water samples (125 mL) were collected during the DeWeX cruises at the surface using Niskin bottles mounted on a CTD frame and immediately fixed with 0.4 mL acidified Lugol (100 g KI, 50 g I, 100 mL glacial acetic acid) and stored at 4 °C. Diatoms were identified and enumerated by light microscopy at X4400 and X8800 magnification depending on size on a Nikon TE-2000 microscope in 50 or 100 mL Utermöhl sedimentation chambers depending on diatom abundance. Large and numerically rare taxa were counted in the entire settling chamber, while smaller dominant taxa such as *Minidiscus* were determined by light microscopy at X 8800 by counting two cuve diameters (i.e., 1/33 of the cuve area), which amounts to a volume of 3 mL counted in the case of 100 mL sedimented samples and 1.5 mL in the case of 50 mL sedimented samples. *Minidiscus trioculatus* and *comicus* were taxonomically determined using an FEI Teneo SEM in one of the very-high abundance samples (station 99, leg 2, 24 April 2013). All similar looking small centrics subsequently observed by light microscopy were assumed to be *Minidiscus*. Data for microscopic diatom counts are available at http://www.obs-vlfr.fr/proof/ftpfree/dewex/db/data/DIATOMS/.

### Semi-continuous automated flow cytometry

Phytoplankton cells were counted semi-continuously (one sample every hour during day time and every 2 h during night time) using an automated Cytosense flow cytometer (Cytobuoy, NL) connected to a continuous seawater flow-through pumped at 3 m depth. In order to ensure an accurate position and limit the distance traveled while analyzing the sample, the Cytosense pumped its sample from an isolated chamber of 300 mL filled in less than 30 s. The chamber was opened to the flow-through in between each analysis. The Cytosense instrument is specially adapted to detecting wide in situ phytoplankton wide size ranges and abundances. Each sample was driven toward the flow cell by a calibrated peristaltic pump running between <1 μL s^−1^ and 20 μL s^−1^ from which the volume analyzed was calculated. Every particle (cell) in suspension in the sample was then separated through a laminar flow thanks to a 0.2 µm filtered sheath fluid made of seawater and crossed a laser beam (Coherent, 488 nm, 20 mW). The instrument recorded various pulse shapes emitted while the cells were crossing the laser beam, resulting in: forward angle light scatter and sideward angle light scatter as well as red, orange, and yellow fluorescence bands in the size range 1–800 µm in width and a few mm in length for chain-forming cells. Laser scattering at small angles was collected by two distinct photodiodes to check for the sample core alignment. Two trigger levels on the red fluorescence were applied for distinction between highly concentrated and picophytoplankton and cyanobacteria groups (trigger level FLR 8 mV, sampling at a flow rate of 10 mL^3^ s^−1^ analyzing ~1 mL), and lower concentrated nano- and microphytoplankton (trigger level FLR 10 mV, at a flow rate of 10 mL^3^ s^−1^ analyzing ~5 mL). Different sets of 2D projections of the data were plotted in Cytoclus® software to manually gate the various phytoplankton groups. A combination of standard beads (PolyScience® Yellow Green Polystyrene 2 μm, 3 μm, 6 μm, 10 μm, and 20 μm diameter) was regularly analyzed to monitor the stability of the flow cytometer. The volume analyzed was weight calibrated.

### Biogeochemical determinations

Biogenic silica samples (2 L) were collected in the same Niskin bottles as cell counts and filtered onto 0.8 µm, 47 mm polycarbonate filters, dried at 60 °C for 24 h, and stored at room temperature. As samples were coastal and susceptible to receive large terrigeneous riverine inputs, they were analyzed in the laboratory following the three-step digestion method allowing for determination of biogenic silica corrected for the interference from lithogenic silica^58^. Data for biogenic silica concentrations are available at http://www.obs-vlfr.fr/proof/ftpfree/dewex/db/data/Si/. POC from water samples was analyzed on GF/F filters on a CHN elemental analyzer. Nutrient samples were stored in 20 mL polyethylene vials and immediately frozen at −20 °C until analysis. In the laboratory, the samples were analyzed by colorimetry on a Seal-Bran-Luebbe AutoAnalyzer AA3 HR^59^.

### Trait-based model of phytoplankton strategists

A trait-based model of a phytoplankton community is used in the present study to explore hypotheses related to the occurrence of the massive *Minidiscus* bloom observed during the DeWeX 2013 campaign. Four types of phytoplankton strategists are represented according to their distinctive traits with regards to the available amounts of light and nutrients. The difference in these physiological traits between phytoplankton types is based on the Reynolds’ C-S-R classification^30^, and combination of these traits within a phytoplankton genus enables to determine its ecological niche. The main assumption of the Reynolds’ model is that a bloom of a given strategist occurs when the environmental conditions (light, nutrients) match its ecological niche. In the present study, the following four strategists are defined. SS-strategists are recurrent nutrient stress tolerant genera in high-light environments, such as the cyanobacteria *Synechococcus* that are constitutive members of the picophytoplankton in the Mediterranean Sea^60^. R-strategists optimally grow in low-light environments, but they require high nutrients in order to form bloom events. This group incorporates many genera of large diatoms with elongated body shapes such as *Chaetoceros, Leptocylindrus*, and *Guinardia*, all of which are genera observed during the spring leg of the DeWeX cruise (see Results section). In our study, R-strategists represent large diatoms with fast sinking rates generally observed during spring blooms at mid-latitudes. The last group is that of C-strategists (colonist genera). The physiological and morphological traits common to all genera of this group are small sizes in the nanoplanktonic spectra, flat cylindrical body shapes, and some fast-growing abilities when nutrients and light are abundant. The choice was made to divide C-strategists into two categories according to close traits: on the one hand, C1-strategists gather autotrophic flagellates and small-sized cryptophyceae that showed a marked presence during the spring leg of DeWeX cruise^61^, on the other hand, C2-strategist represents *Minidiscus* diatoms.

The two latter strategists, C1 and C2, are considered to be in the same range of size and to have similar traits^62^. In our model, some differences in traits are, however, assumed and they go beyond just a difference in a reliance or not on Si uptake for the C2- and C1-strategists. In particular, the C2-strategist is also considered more tolerant to light stress and less tolerant to nutrient stress than C1-strategists. These different abilities are based on knowledge of the spring succession of the phytoplankton community acquired by remote sensing^63^ and in situ observations^64^ in the northwestern Mediterranean Sea. These studies indicate that the first community to peak is generally diatoms from January to March followed several weeks later by a peak of nano-eukaryotes at the beginning of the stratification period when surface nutrients decrease. This is the usual pattern of the second stage of the bloom dominated by nano-eukaryotes (i.e., autotrophic flagellates, small cryptophyceae) that has typically been observed in various oceanic areas at mid-latitudes^65,66^. As suggested in the study of Marty et al. (2002)^64^, nano-eukaryotes would flourish during the time period for which successive events of stratification and destratification of the water column accompanied with weak injections of nutrients from deep layers occur. These differences in traits involve some emergent properties specific to each strategist such as, for example, their ability to grow differently depending on light and nutrient availability (see net photosynthetic growth rates in Supplementary Table 5). The choices of parameters in the trait-based model are crucial because they determine in fine the differences in functional traits between strategists. These choices have been made on the basis of different sources for usual allometric rules between functional traits and size or biovolume^67,68^ as well as different types of experimental data (field, laboratory assays). All the sources of parameters are indicated in the Suppl. Tables 1–4). For example, in the model, the half-saturation constant for nitrogen uptake increases with cell size while the photosynthetic efficiency (given in our model by the product of maximum quantum yield by Chl-specific absorption coefficient) decreases along cell size. In the same way, the choices of values for internal quotas are based on the observations of decreasing stoichiometric molar quotas along increasing cell volumes^23,69^ and from the use of the combined dataset of carbon cell contents vs. cell volume^68,70^.

However, when a new strategist (nano-diatoms in the present study) is incorporated in a trait-based model, it can often be difficult to allocate an accurate value to each of its physiological parameters owing to insufficient experimental information. In this case, the choices of the latter parameters are rather made on a qualitative idea that the trait of the strategist should be affected by environmental conditions. The difference in mortality rates between C1- and C2-strategists illustrates this point: the C1-strategist is assumed to represent a heterogeneous mix of nano-eukaryotes while the C2-strategist is a numerical representation of closely related species of one genus (i.e., *Minidiscus*). It is hypothesized that the mortality processes would have lower impacts on a heterogeneous community of plankton species because if one species collapses under a viral attack, for example, another one will arise, which enables the maintenance of numerous nano-eukaryotes. This mechanism is less likely to occur when a more homogeneous group such as *Minidiscus* is considered.

The type of trait-based model used here has been implemented in the biogeochemical modular numerical platform Eco3M^71,72^. The main characteristics of the model are mentioned hereafter. Each group of strategists is represented through several states variables of C, N, P, Si, and Chl contents and intracellular ratios can thus be computed at each time point allowing a non-redfieldian behavior of the model. They have the ability to take up dissolved inorganic and organic matter (i.e., mixotrophy) and to exude organic matter in order to adjust their stoichiometric internal requirements. A heterotrophic bacteria compartment is also considered for their ability to recycle organic matter into inorganic nutrients. Compartments of inorganic nutrients such as nitrate, phosphate, and silicic acid, as well as of organic matter are represented. Grazers and viruses of phytoplankton are not explicitly accounted for in the model but their processes of control are implicitly represented through mortality rates. All the mathematical formulations of processes are provided in details in Campbell et al.^73^.

Two numerical experiments were conducted to test the impact of these processes on the temporal dynamics of the different strategists. In particular, the mortality rate of R-strategists has been thus modulated upward (HCC: High Control Conditions) or downward (LCC: Low Control Conditions, standard simulation). In the HCC condition, the mortality rate corresponds to a disappearance of biomass by 25% d^−1^ while this rate is 10% d^−1^ in the LCC condition according to the study of Broglio et al.^74^. Given the short simulation time (40 days) variations in water temperature are neglected and no temperature-dependent processes are then considered in the model formulations. This assumption is corroborated by field observations that show temperature variation lower than 1 °C over the time of the simulated period^21^.

Model parameters characterizing each group of strategists and initial conditions in nutrients, living biomasses and organic matter are given in Supplementary Table 1. The standard run is launched from initial conditions in nutrients corresponding to those observed during DeWeX^75^ just at the end of the high convective episode (HCNC: High Convective Nutrient Condition) with [H_4_SiO_4_] = 7.72 µM, [NO_3_] = 8.40 µM, and [PO_4_] = 0.39 µM. Another run is launched with a reduced supply of nutrients characterizing reduced convective events (LCNC: Low Convective Nutrient Condition). These concentrations were chosen in the PERSEUS database for a low convective year (1990) and were set at [H_4_SiO_4_] = 2.10 µM, [NO_3_] = 2.66 µM, and [PO_4_] = 0.09 µM.

All simulations for the present study were performed under a constant irradiance of 300 W m^−2^ and a 12 h/12 h day/night cycle corresponding to the beginning of spring at mid-latitudes. Time step of the model is 300 s. The time of simulation is 40 days (roughly corresponding to the period of bloom during DeWeX) from the end^27^. From this last event, it is assumed that a light vs. nutrient optimal tradeoff exists for the phytoplankton bloom without any further variation of the physical environment (e.g., nutrient supplies, light variations). In this theoretical context, a trait-based model without considering any physical forcing (0D) can be used over the simulation period. Although useful to understand community dynamics, it does limit the model’s ability to correctly estimate in situ biomass levels because no loss terms due to physics are included, while considerable turbulent mixing and advection are known to occur in the area. Model parameters and initial conditions of state variables are presented in Supplementary Tables 1–5.

### Sediment trap data

A short mooring line (~50 m long) was deployed at 42° 01′N–4° 48′E (depth of 2400 m). It was equipped with a Technicap PPS-3 sediment trap (collecting area of 0.125 m^2^, aspect ratio of 2.5, and 12 collecting cups) at 30 m above the seabed. The trap samples were collected with sampling interval between 15 and 23 days. Prior to deployment, the sampling bottles were filled with 0.45 µm filtered seawater containing sodium borate-buffered formalin to yield a final concentration of 5% formalin to prevent in situ microbial decomposition. Upon recovery, samples were stored in the dark at 4 °C^76^. 1 mL aliquots were filtered onto the center of 0.4 µm polycarbonate filter using a filtering funnel of 6 mm aperture, and carefully rinsed with DIW water, then dehydrated in increasing series of ethanol 30%, 50%, 70%, 80%, 90%, and 100% during 10 mn for each step. The samples were completely dried overnight, mounted on aluminum stubs with double sticky carbon tabs, and sputter-coated with gold for 10 min. The samples from each of the three available trap samples were analyzed with a FEI Teneo SEM.

### *Tara* Oceans V9-18S rDNA metabarcodes

We used the global metabarcoding dataset (EBI accession number PRJEB16766) generated from the biological samples collected from 146 sampling locations during the *Tara* Oceans expedition^12,77,78^. We extracted ribotypes that were assigned to *Minidiscus* and *Minutocellus* from the three depths—surface, deep chlorophyll maximum, and MESO—within the different size-class filters (ranging from 0.8 to 2,000 µm, with the smallest fraction being 0.8 to 5 µm). The taxonomic assignations were done using PR2 reference database^79^ which has six reference sequences from *Minidiscus trioculatus*, 2 from *Minidiscus* sp., and one from an environmental sequence. From the photic zone, for *Minidiscus*, a total of 908 different V9 rDNA ribotypes (represented by 66,043 reads) were retrieved from the 81 communities representing the smallest size-fraction (0.8 to 5 µm), while for *Minutocellus* we retrieved 776 V9 rDNA ribotypes (represented by 35,108 reads) from the 118 communities representing the smallest size-fraction (0.8–5 µm). From the mesopelagic zone, a total of 49,239 reads for *Minidiscus* and 27,181 reads for *Minutocellus* were retrieved from 79 size-fractionated samples. Relative abundance was calculated with respect to total diatom reads and to total phytosynthetic reads, which comprised reads assigned to major phytoplanktonic groups^80^, namely Bacillariophyta, Chlorophyceae, Cryptophyta, Dictyochophyceae, Dinophyceae, Haptophyta, Mamiellophyceae, Pelagophyceae, and Raphidophyceae. Exponentiated Shannon-Weiner H’ diversity index was used as an estimate of diversity at each station. All the analyses were conducted using open source R version 3.3.1 (data and R-script available at https://figshare.com/s/6f3190905564f6c6e20c).

### Data availability

The authors declare that all data supporting the findings of this study are available within the paper and its Supplementary Information files.

## Electronic supplementary material

Supplementary Information
