# Supplementary material for: Nanoplanktonic diatoms are globally overlooked but play a role in spring blooms and carbon export
Source: Nat Commun. 2018 Mar 5;9:953. doi: 10.1038/s41467-018-03376-9 (PMC5838239; doi:10.1038/s41467-018-03376-9)
Supplement: Supplementary file 1 — Supplementary Information [file 41467_2018_3376_MOESM1_ESM.pdf]

Nanoplanktonic diatoms are globally overlooked but play a role in spring blooms and carbon export

Leblanc et al.

Supplementary Information

SUPPLEMENTARY FIGURES

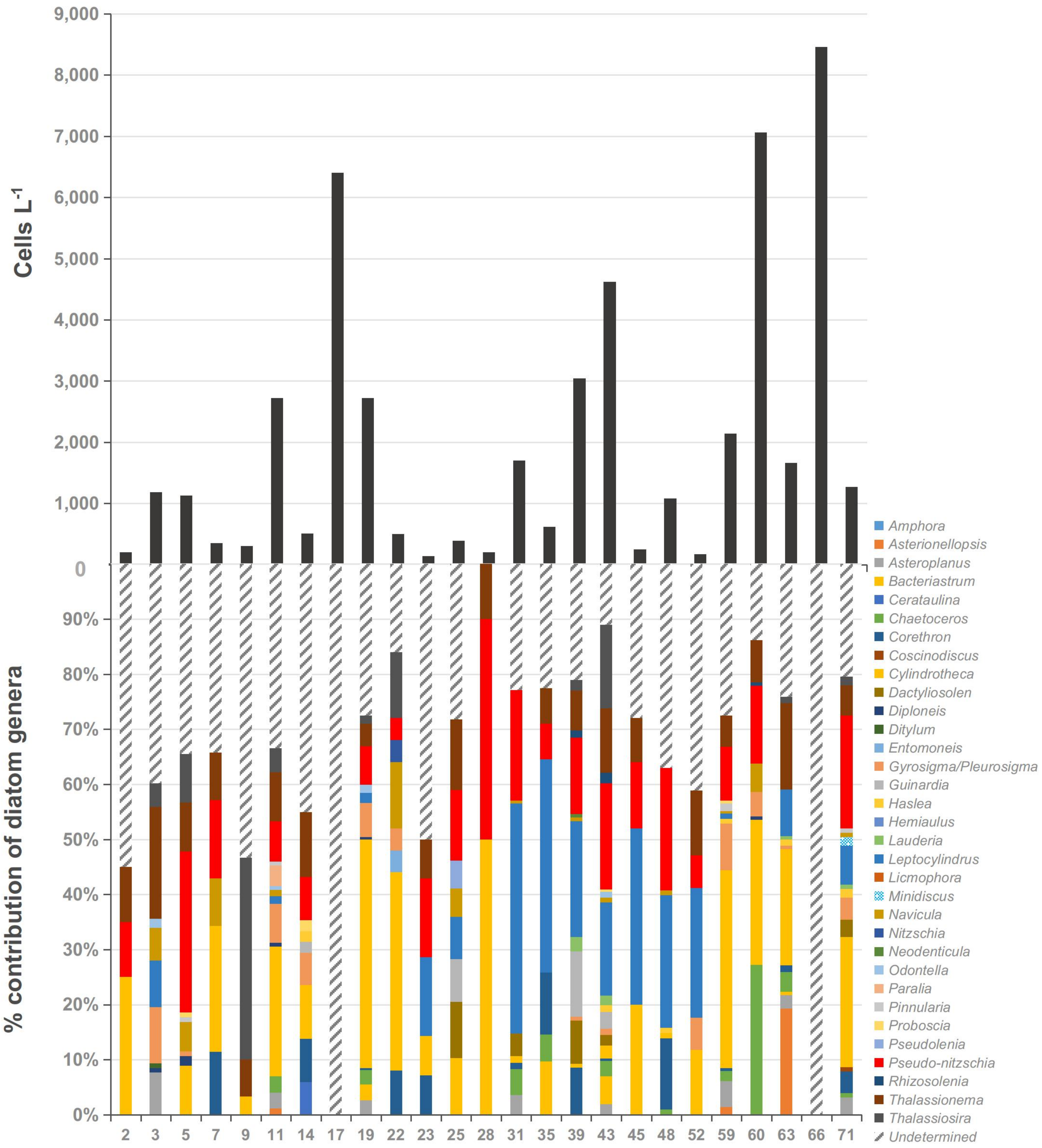

Supplementary Figure 1: **Total diatom abundance and relative genera contribution during DeWeX leg 1.** Grey bars on the top panel are diatom abundance in cells L^{-1} while the lower panel indicates the relative contribution of each genera in %. Station numbers correspond to sampling stations identified in Fig.1a. Note that *Minidiscus* only appears in very low abundance at station 71 at the end of leg 1.

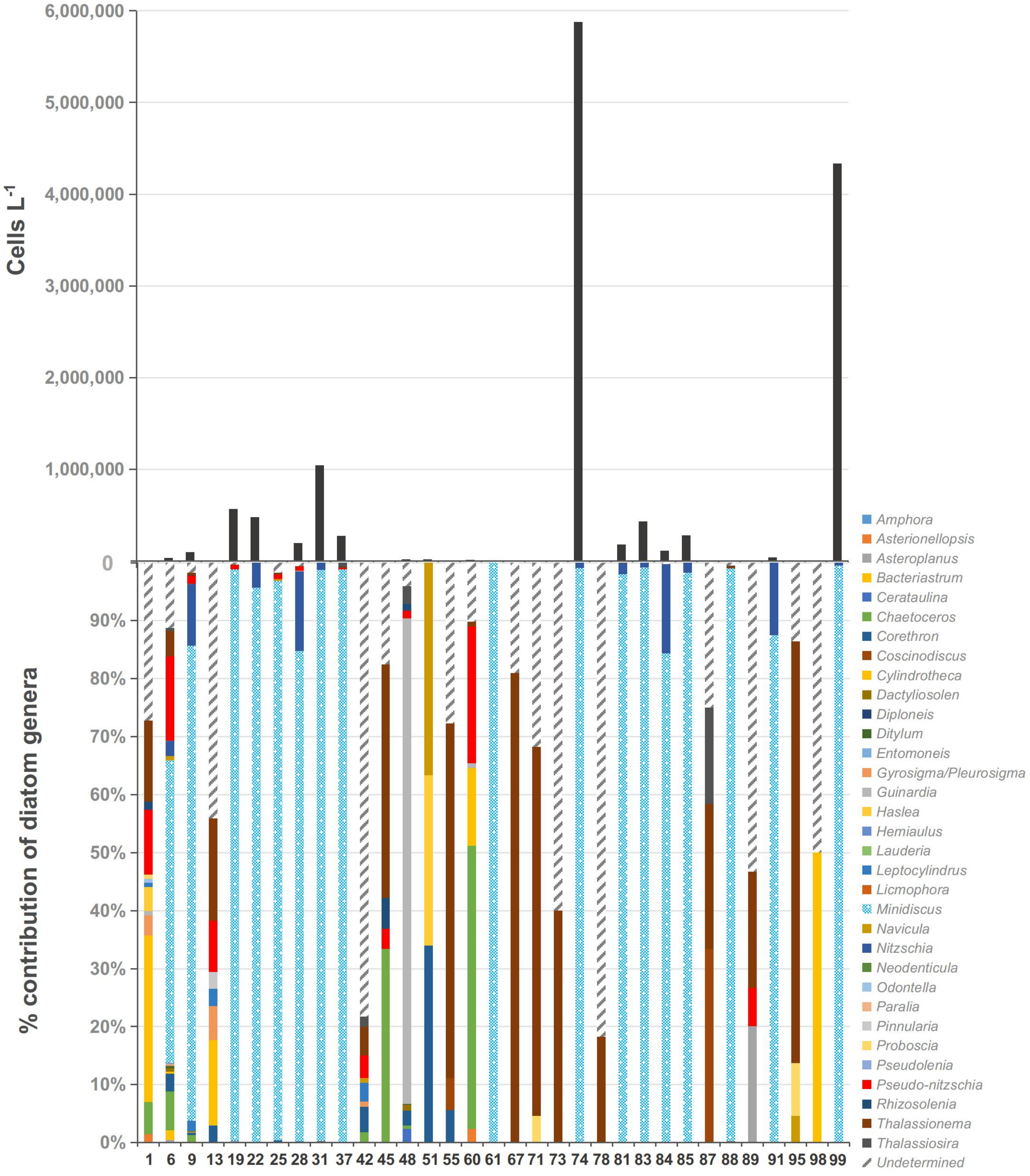

Supplementary Figure 2 : **Total diatom abundance and relative genera contribution during DeWeX leg 2.** Grey bars on the top panel are diatom abundance in cells L^{-1} while the lower panel indicates the relative contribution of each genera in %. Station numbers correspond to sampling stations identified in Fig.1b. Note that *Minidiscus* is largely dominant at 17 stations, mostly where diatom abundance is high.

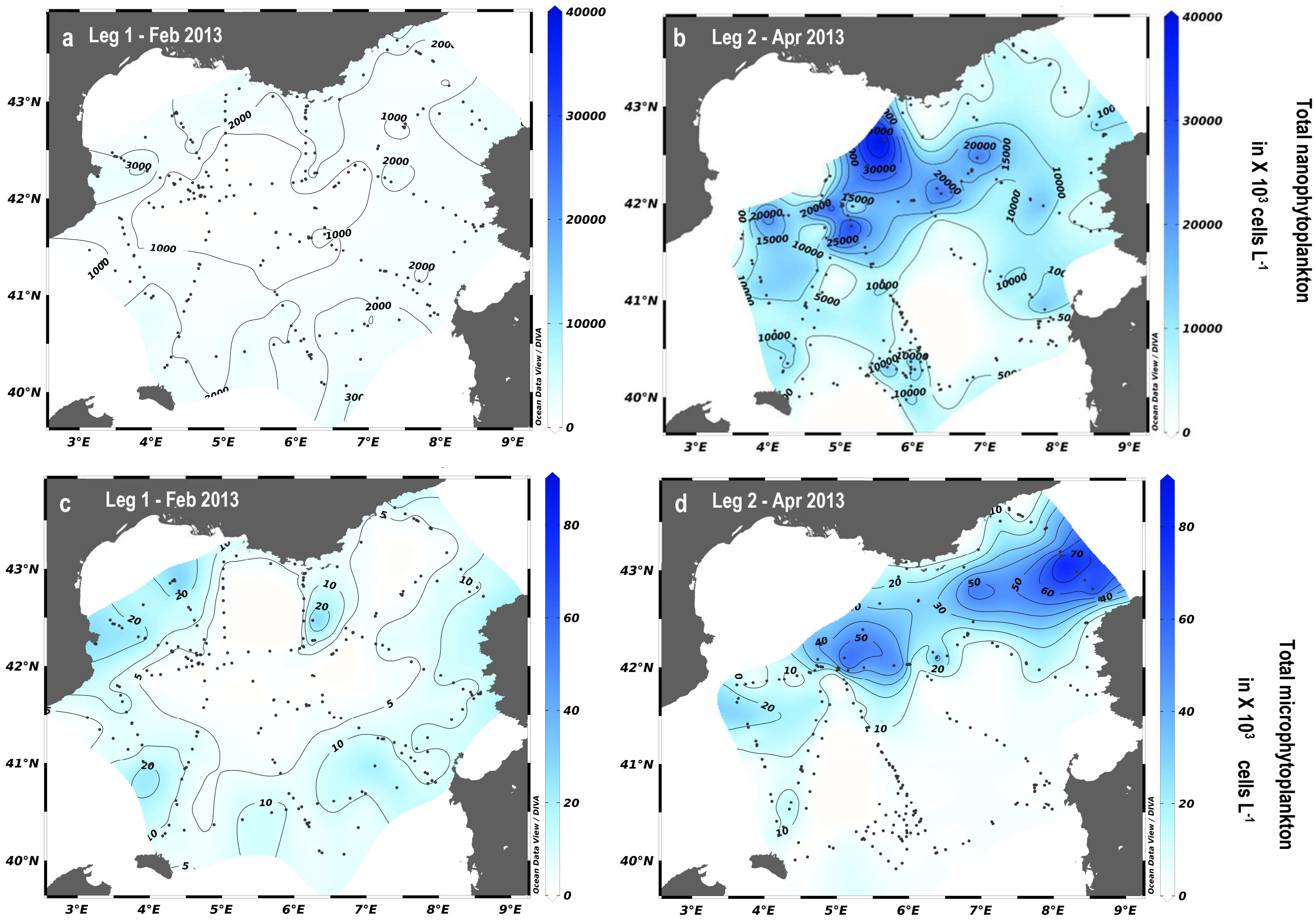

Supplementary Figure 3: **Total nanophytoplankton and microphytoplankton abundances determined by flow cytometry during DEWEX.** Surface distribution of nanophytoplankton during a. leg 1 (03-21 February 2013) and b. leg 2 (05-24 April 2013) of the DEWEX cruise, and of microphytoplankton during c. leg 1 and d. leg 2. Cell abundances in x10^3 cells L^{-1} were obtained using an automated flow cytometer installed on a seawater continuous pumping system.

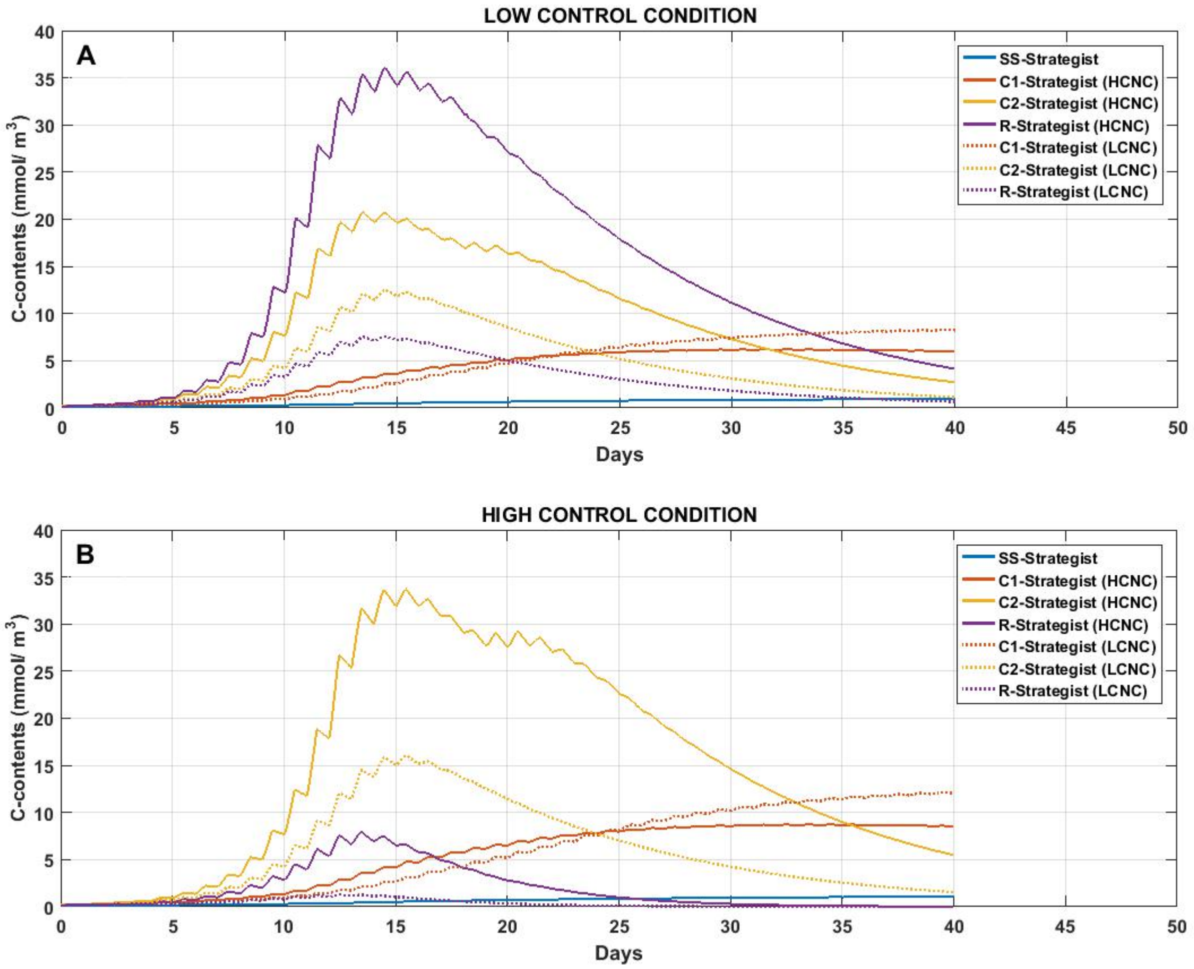

Supplementary Figure 4: **Simulated succession of C-S-R strategists during the DeWeX spring bloom.** Model outputs are based on the Eco3M-platform following the simulation detailed in the Method section. (A) Succession of different plankton strategists during high and low convective nutrient conditions (HCNC and LCNC, respectively) but with low control condition (LCC) on R-strategists. (B) Same groups with high control condition (HCC) on R-strategists. The model simulation closest to DeWeX conditions is displayed in the B panel in HCC and HCNC, and correctly simulates the dominance of C2-strategists over R-strategists and high levels of biomass.

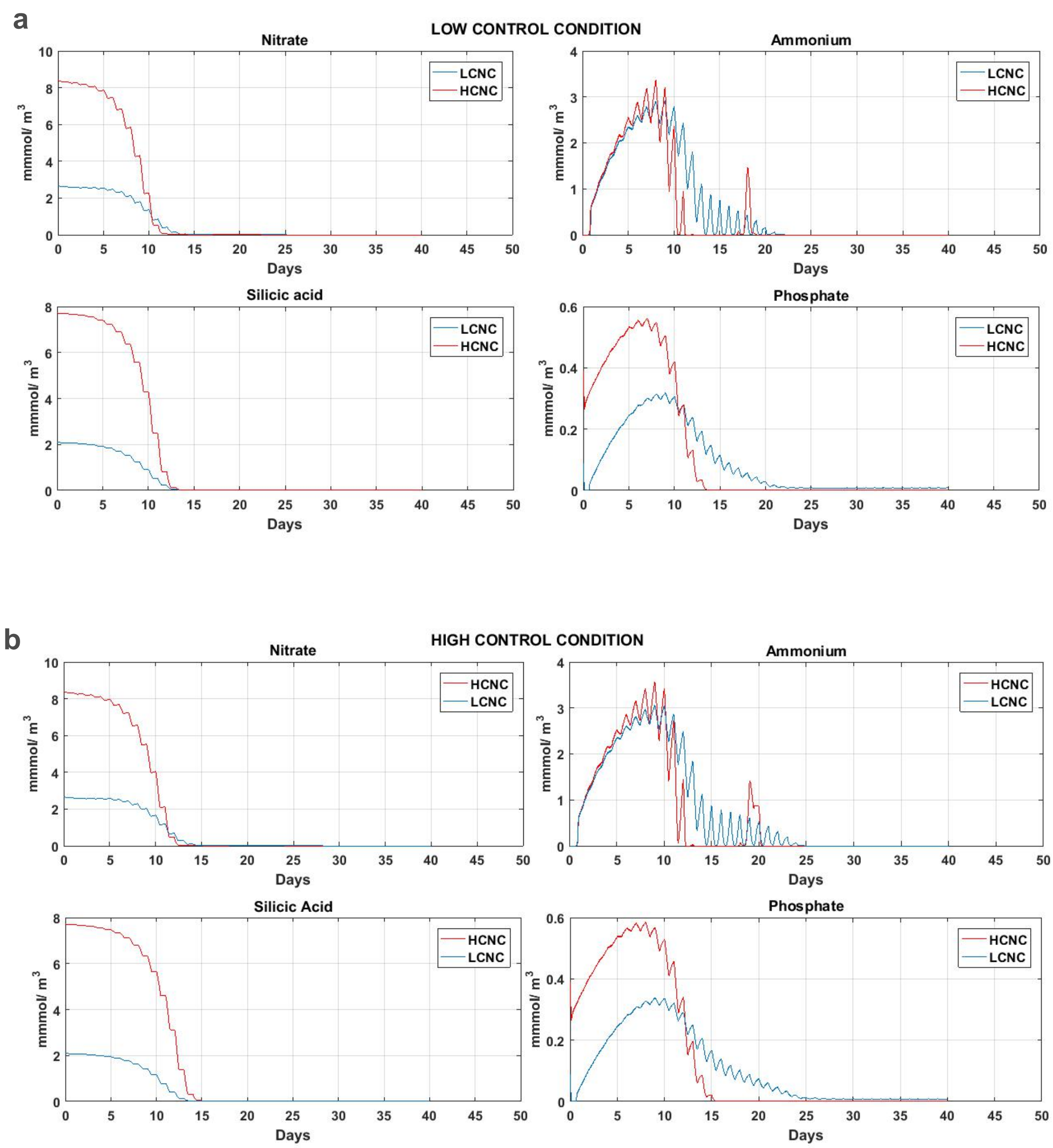

Supplementary Figure 5: **Simulated succession of nutrients during the DeWeX spring bloom.** Model outputs are based on the Eco3M-platform following the simulation detailed in the Method section. (a) and (b) Temporal evolution of nutrients during LCC and HCC, respectively.

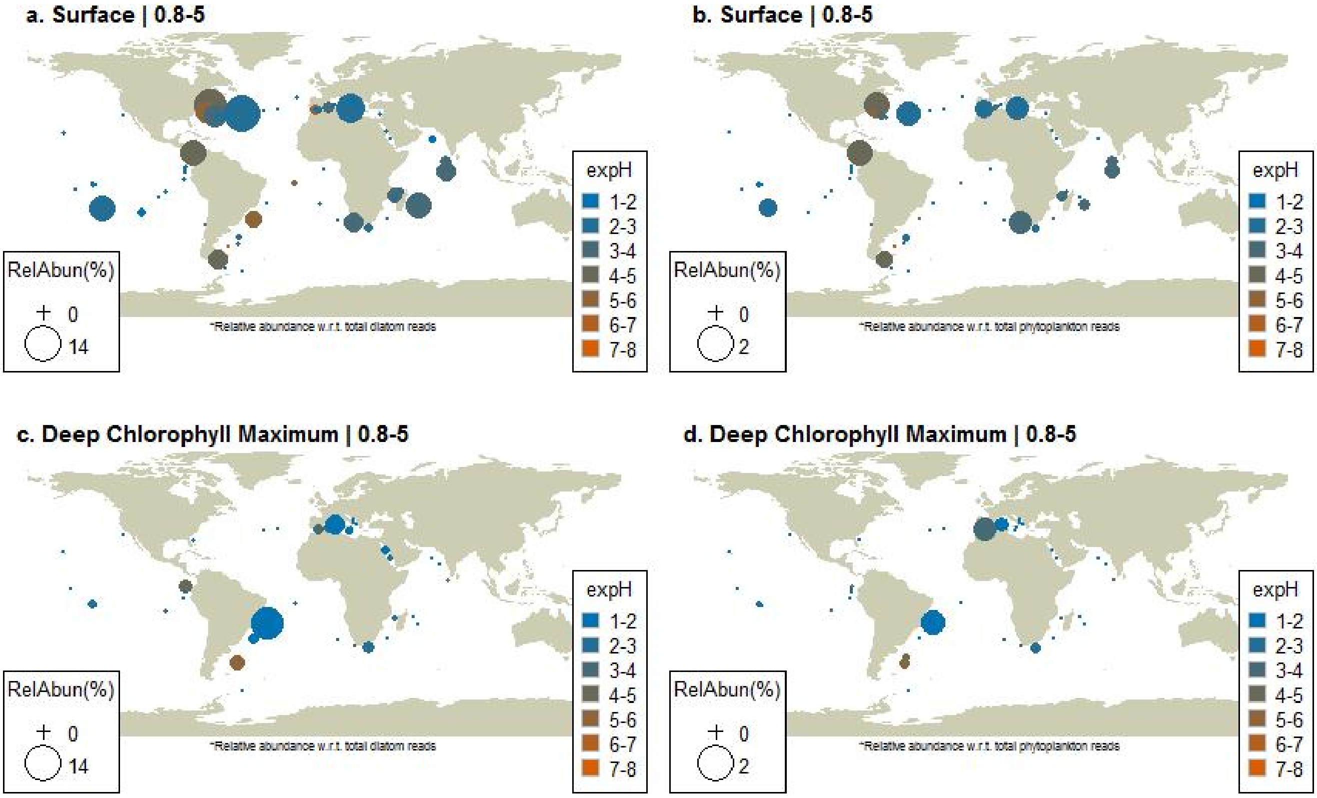

Supplementary Figure 6: **Biogeographical distributions of *Minutocellus* from metabarcoding data.** Biogeographical distributions at the surface and DCM (Deep Chlorophyll Maximum) depths of genus abundance and diversity of *Minutocellus* as relative abundance of total diatom reads (a-c) and as relative abundance of total phytoplankton reads (b-d) in the 0.8 to 5 μm size fractions collected during the *Tara* Oceans expedition (2009-2013). The variation in diversity for each genus is indicated as the exponentiated Shannon Diversity Index (expH) and the color represents the number of unique ribotypes (blue= low richness; orange= high richness). Bubble symbols are scaled to indicate the relative percent reads of each genus with respect to total diatoms (a-c) or total photosynthetic reads in the samples (b-d).

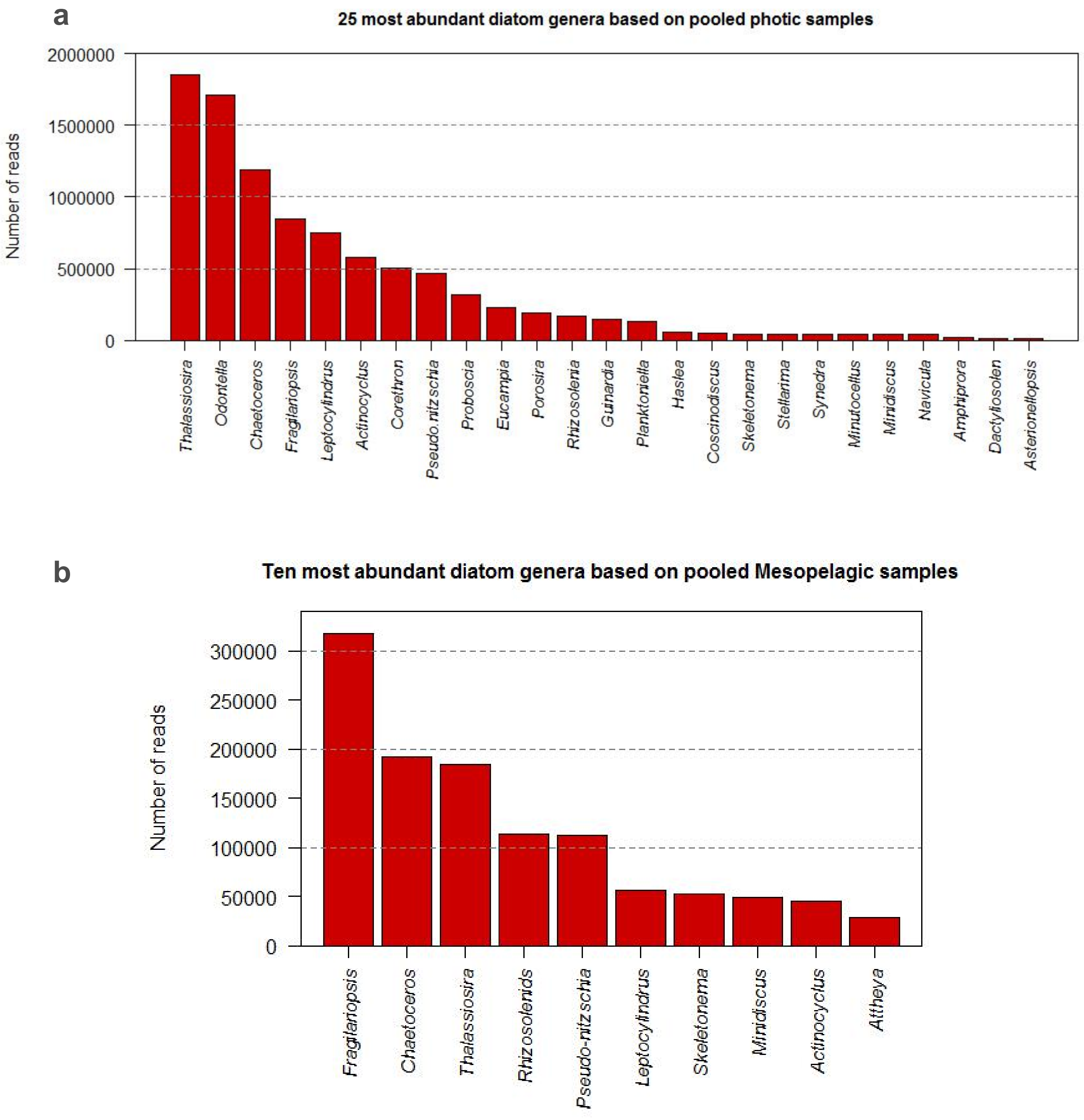

Supplementary Figure 7: **Relative ranks of *Minidiscus* and *Minutocellus* in pooled mesopelagic and photic zone samples.** *Minutocellus* and *Minidiscus* are the 20th and 21st most abundant diatom genera in photic zone samples (a) and *Minidiscus* is the 8th most abundant diatom genus in mesopelagic samples (b) from the *Tara* Oceans data set.

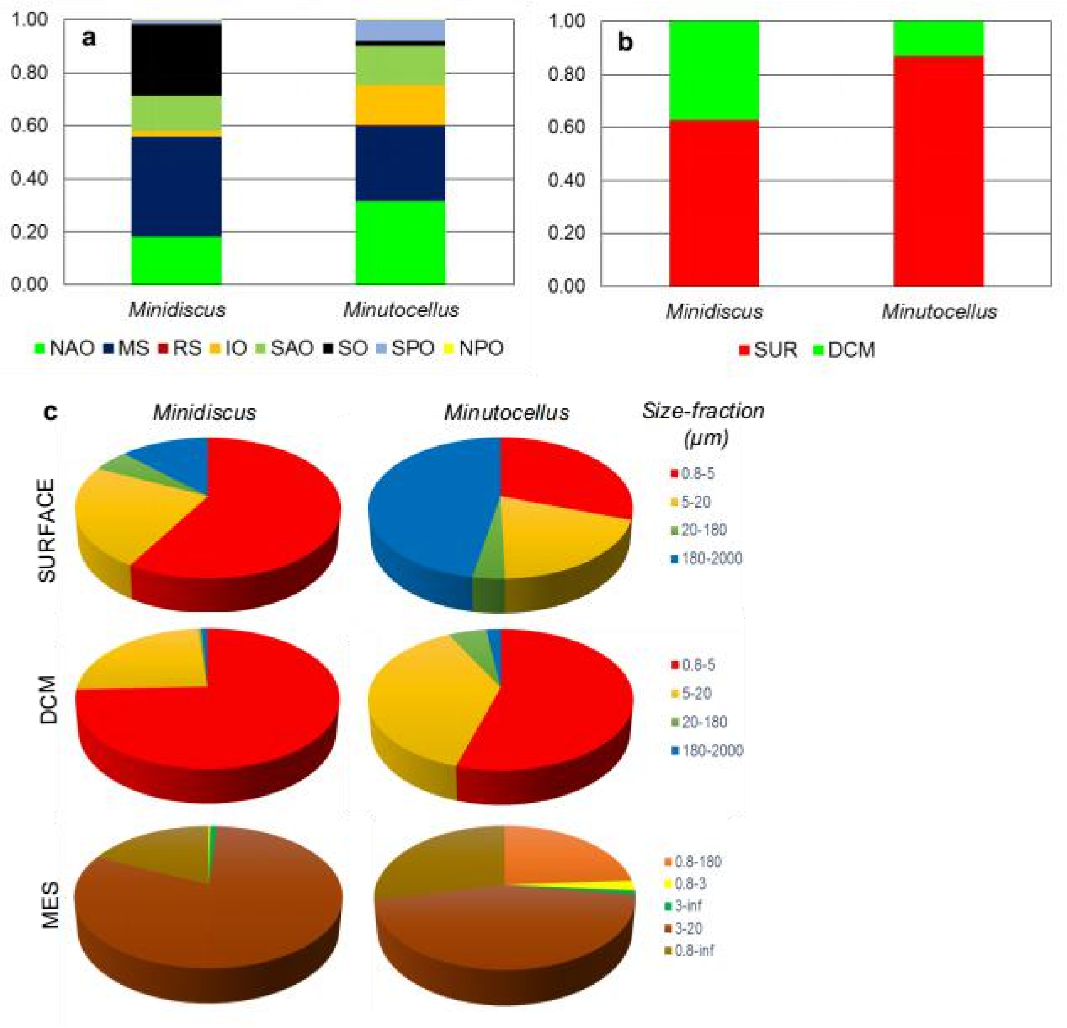

Supplementary Figure 8: **Relative distributions of the diatom genera *Minidiscus* and *Minutocellus*.** (a) Abundances derived from the 0.8 to 5 µm size-fraction from *Tara* Oceans. NAO: North Atlantic Ocean, MS: Mediterranean Sea, RS: Red Sea, SAO: South Atlantic Ocean, IO: Indian Ocean, SO: Southern Ocean, SPO: South Pacific Ocean, NPO: North Pacific Ocean. (b) Relative distribution of *Minidiscus* and *Minutocellus* in 0.8 to 5 µm size-fraction surface and Deep Chlorophyll Maximum (DCM) fractions. (c) Relative distribution by size fractions (in µm) at the surface, DCM and in mesopelagic samples (MES) collected at an average of 700 m.

SUPPLEMENTARY TABLES

Phytoplankton	Unit	R-strategist	C1-strategist	C2-strategist	SS-strategist	Ref
Max. quantum yield	mmolC J^{-1}	$2.55E^{-4}$	$1.80E^{-4}$	$2.00E^{-4}$	$1.64E^{-4}$	*1,2,3*
Chl-specific absorption coeff.	m^2 mg^{-1}	0.015	0.030	0.025	0.050	*1,2*
Photosystems renewal time	d	$2.30E^{-8}$	$5.44E^{-8}$	$2.30E^{-8}$	$8.10E^{-8}$	*4*
Photosystems cross-section	m^2 J^{-1}	10.9	15.8	13.7	21.0	*5,6*
PSII damage rate	-	$2.6E^{-8}$	$2.6E^{-8}$	$2.6E^{-8}$	$2.6E^{-8}$	*7*
Rate of repair of damaged PSII	d^{-1}	$2.0E^{-9}$	$2.0E^{-9}$	$2.0E^{-9}$	$2.0E^{-9}$	*7*
Min. internal N/C quota	molN $molC^{-1}$	0.050	0.100	0.070	0.115	*8,9,10,11*
Max. internal N/C quota	molN $molC^{-1}$	0.170	0.215	0.180	0.229	*9,10,11,12,13*
Min. internal P/C quota	molP $molC^{-1}$	0.0031	0.0062	0.0044	0.0071	*10,11,14,15*
Max. internal P/C quota	molP $molC^{-1}$	0.0100	0.0130	0.0110	0.0143	*10,11,14,15*
Min. internal Si/C quota	molSi $molC^{-1}$	0.040	-	0.056	-	*1,15*
Max. internal Si/C quota	molSi $molC^{-1}$	0.136	-	0.144	-	*1,15*
Max. internal Chl/N quota	molChl $molN^{-1}$	3.00	2.55	2.70	2.20	*16,17,18*
Respiration cost for growth	-	0.20	0.30	0.20	0.32	*17,19*
Half sat. constant for NO_3	mmolN m^{-3}	3.50	1.50	1.75	0.73	*8,15,20,21*
Half sat. constant for NH_4	mmolN m^{-3}	0.18	0.12	0.15	0.07	*8,20,21*
Half sat. constant for PO_4	mmolP m^{-3}	0.200	0.055	0.070	0.008	*8,15,20,22*
Half sat. constant for silicic acid	mmolSi m^{-3}	2.75	-	1.20	-	*8,15*
Constant in the quota function for silicic acid uptake	molSi $molC^{-1}$	0.10	-	0.10	-	*23*
Shape constant in the quota function for silicic acid uptake	-	10.	-	10.	-	*23*
Half sat. constant for DON	mmolN m^{-3}	2.25	1.50	2.05	0.85	*8,21*
Half sat. constant for DOP	mmolP m^{-3}	0.65	0.155	0.55	0.085	*8,22*
Resp. cost for NO_3 uptake	molC $molN^{-1}$	0.397	0.397	0.397	0.397	*19*
Resp. cost for NH_4 uptake	molC $molN^{-1}$	0.198	0.198	0.198	0.198	*20*
Resp. cost for PO_4 uptake	molC $molP^{-1}$	0.350	0.350	0.350	0.350	*21*
Resp. cost for silicic acid uptake	molC $molSi^{-1}$	0.140	-	0.140	-	*22*
Mortality rate	d^{-1}	0.10 , 0.25(HCC)	0.02	0.10	0.05	*24,25,26*

Supplementary Table 1: **Numerical parameters of the trait-based model.**

Heterotrophic bacteria	Unit	Value	Ref
Maximum growth rate	d^{-1}	1.20	*27*
Half-sat. for DOC uptake	mmolC m^{-3}	50	*28*
Half-sat. for DON uptake	mmolN m^{-3}	1.50	*27*
Half-sat. for DOP uptake	mmolP m^{-3}	0.080	*29*
Half-sat. for NH_4 uptake	mmolN m^{-3}	0.150	*28*
Half-sat. for PO4 uptake	mmolP m^{-3}	0.020	*29*
Min. internal N/C quota	molN $molC^{-1}$	0.168	*30*
Max. internal N/C quota	molN $molC^{-1}$	0.264	*30*
Min. internal P/C quota	molP $molC^{-1}$	0.0083	*30*
Max. internal P/C quota	molP $molC^{-1}$	0.0278	*30*
Mortality rate	d^{-1}	0.12	*27*

Supplementary Table 2 : **Numerical parameters of the trait-based model.**

Non-living matter	Unit	Value	Ref
C detritus remineralisation rate	d^{-1}	0.006	*28*
N detritus remineralisation rate	d^{-1}	0.005	*28*
P detritus remineralisation rate	d^{-1}	0.010	*29*
Detritus remineralisation rate, Si	d^{-1}	0.0003	*24*
Nitrification rate	d^{-1}	0.050	*24*

Supplementary Table 3 : **Numerical parameters of the trait-based model.**

**Phytoplankton**	**Unit**	**R-strategist**	**C1-strategist**	**C2-strategist**	**SS-strategist**
Carbon biomass	mmolC m^{-3}	0.156	0.200	0.175	0.100
Nitrogen biomass	mmolN m^{-3}	0.024	0.030	0.026	0.015
Phosphorus biomass	mmolP m^{-3}	0.0015	0.0019	0.0017	0.0009
Biogenic silica content	mmolSi m^{-3}	0.019	-	0.020	-
Chlorophyll biomass	mg m^{-3}	0.038	0.048	0.042	0.024
**Heterotrophic bacteria**					
Carbon biomass	mmolC m^{-3}	30			
Nitrogen biomass	mmolN m^{-3}	7			
Phosphorus biomass	mmolP m^{-3}	0.66			
**Inorganic Nutrients**		**HCNC**	**LCNC**		
Nitrate	mmolN m^{-3}	8.40	2.66		
Ammonium	mmolN m^{-3}	0.001	0.004		
Phosphate	mmolP m^{-3}	0.39	0.09		
Silicic acid	mmolSi m^{-3}	7.72	2.10		
**Dissolved organic matter**					
Carbon	mmolC m^{-3}	7			
Nitrogen	mmolN m^{-3}	1.06			
Phosphorus	mmolP m^{-3}	0.066			
**Particulate organic matter**					
Carbon	mmolC m^{-3}	$0.665E^{-3}$			
Nitrogen	mmolN m^{-3}	$0.100E^{-3}$			
Phosphorus	mmolP m^{-3}	$0.627E^{-5}$			
Silicon	mmolSi m^{-3}	$0.665E^{-4}$			

Supplementary Table 4: **Initial conditions for the state variables of the trait-based model under high and low convective nutrient conditions (HCNC and LCNC, respectively).**

Low Control Conditions (LCC)	10th day	15th day	30th day	High Control Conditions (HCC)	10th day	15th day	30th day
**HCNC**				**HCNC**			
**SS-strategist**	0.36	0.19	-0.03	**SS-strategist**	0.36	0.29	-0.03
**C1-strategist**	2.28	0.87	0.14	**C1-strategist**	2.24	1.15	0.16
**C2-strategist**	2.86	0.15	-0.29	**C2-strategist**	2.87	0.46	-0.28
**R-strategist**	3.11	0.33	-0.30	**R-strategist**	3.01	0.22	-0.78
**LCNC**				**LCNC**			
**SS-strategist**	0.29	0.29	-0.01	**SS-strategist**	0.30	0.29	-0.01
**C1-strategist**	2.15	1.50	0.22	**C1-strategist**	2.12	1.75	0.25
**C2-strategist**	2.60	0.30	-0.34	**C2-strategist**	2.67	0.53	-0.34
**R-strategist**	2.39	0.25	-0.36	**R-strategist**	2.24	-0.10	-0.89

Supplementary Table 5: **Net photosynthetic rates of modeled strategists during the simulations.** Values of net photosynthetic growth rates (d^{-1}) of each strategist at three different times (at noon on the 10th, 15th and 30th days) of the simulation in Low Control condition (LCC) and High Control condition (HCC), under high and low convective nutrient conditions (HCNC and LCNC respectively). The net growth rates of all strategists are realistic and within the orders of magnitude of the observed datasets[1,15,31].

SUPPLEMENTARY REFERENCES

1. Terseleer, N., Bruggeman, J., Lancelot, C. & Gypens, N. Trait-based representation of diatom functional diversity in a plankton functional type model of the eutrophied southern North Sea. *Limnol. and Oceanogr.* **59**, 1958-1972 (2014).
2. Claustre, H. *et al.* Toward a taxon-specific parameterization of bio-optical models of primary production: a case study in the North Atlantic. *J. Geophys. Res.* ***110***, C07S12 (2005).
3. Babin, M. *et al.* Nitrogen- and irradiance-dependent variations of the maximum quantum yield of carbon fixation in eutrophic, mesotrophic and oligotrophic marine systems. *Deep Sea Res. I* **43**, 1241–1272 (1996).
4. Laney, S., Letelier, R. & Abbott, M. Parameterizing the natural fluorescence kinetics of *Thalassiosira weissflogii*. *Limnol. and Oceanogr.* **50**, 1499-1510 (2005).
5. Gorbunov, M., Kolber, Z. & Falkowski, P. Measuring photosynthetic parameters in individual algal cells by Fast Repetition Rate fluorometry. *Photosynth. Res.* **62**, 141-153 (1999).
6. Moore, C.M. *et al.* Physical controls on phytoplankton physiology and production at a shelf sea front: a fast repetition rate fluorometer based field study. *Mar. Ecol. Progr. Ser.* **259**, 29-45 (2003).
7. Oliver, R.L., Whittington, J., Lorenz, Z. & Webster, I.T. The influence of vertical mixing on the photoinhibition of variable chlorophyll a fluorescence and its inclusion in a model of phytoplankton photosynthesis. *J. Plankton Res.* **25**, 1107-1129 (2003).
8. Lichtman, E., Klausmeier, CA., Schofield, OM. & Falkowski, PG. The role of functional traits and trade-offs in structuring phytoplankton communities: scaling from cellular to ecosystem level. *Ecol. Letters* **10,** 1170-1181 (2007).
9. Heldal, M., Scanlan, D.J., Norland, S., Thingstad, F. & Mann, N.H. Elemental composition of single cells of various strains of marine *Prochlorococcus* and *Synechococcus* using X-ray microanalysis. *Limnol. and Oceanogr.* **48**, 1732-1743 (2003).
10. Riegman, R., Stolte, W., Noordeloos, A. & Slezak, D. Nutrient uptake and alkaline phosphatase (EC 3:1:3:1) activity of *Emiliania huxleyi* (Prymnesiophyceae) during growth under N and P limitation in continuous cultures. *J. Phycol.* **36(1)**, 87-96 (2000).
11. Geider, R.J., Macintyre, H.L., Graziano, L.M. & McKay, R.M.L. Responses of the photosynthetic apparatus of *Dunaliella tertiolecta* (Chlorophyceae) to nitrogen and phosphorus limitation. *Eur. J. Phycol.* **33**, 315-332 (1998).
12. Menden-Deuer, S. & Lessard, E.J. Carbon to volume relationships for dinoflagellates, diatoms, and other protist plankton. *Limnol. and Oceanogr.* **45**, 569-579 (2000).
13. Mullin, M.,M., Sloan, P.,R. & Eppley, R.,W. Relationship between carbon content, cell volume, and area Inphytoplankton. *Limnol. and Oceanogr.* **11(2)**, 307-311 (1966).
14. Bertilsson, S., Berglund, O., Karl, D. & Chisholm, S. Elemental composition of marine *Prochlorococcus* and *Synechococcus*: implications for the ecological stoichiometry of the sea. *Limnol. and Oceanogr.* **48**, 1721-1731 (2003).
15. Sarthou, G., Timmermans, K., Blain, S. & Tréguer, P. Growth physiology and fate of diatoms in the ocean: a review. *J. Sea Res.* **53**, 25-42 (2005).
16. Geider, R.J., MacIntyre, H.L. & Kana, T.M. Dynamic model of phytoplankton growth and acclimation: Responses of the balanced growth rate and the chlorophyll a: carbon ratio to light, nutrient-limitation and temperature. *Mar. Ecol. Progr. Ser.* **143**, 187-200 (1997).

17. Nielsen, M., 1997. Growth, dark respiration and photosynthetic parameters of the coccolithophorid *Emiliania huxleyi* Prymnesiophyceae) acclimated to different day length-irradiance combinations. *J. Phycol.* **33**, 818-822.
18. Moore, J.K., Doney, S.C &, Lindsay, K. Upper ocean ecosystem dynamics and iron cycling in a global three-dimensional model. *Glob. Biogeochem. Cycles* **18**, GB4028 (2004).
19. Thornley, J. & Cannell, M. Modelling the components of plant respiration: representation and realism. *Annals of Botany* **85**, 55-67 (2000).
20. Harrison, W., Harris, L. & Irwin, B. The kinetics of nitrogen utilization in the oceanic mixed layer: nitrate and ammonium interactions at nanomolar concentrations. *Limnol. and Oceanogr.* **41(1)**, 16-32 (1996).
21. Tyrrell, T. & Taylor, A.A modelling study of *Emiliania huxleyi* in the NE atlantic. *J. Mar. Sys.* **9**, 83-112 (1996).
22. Timmermans, K., der Wagt, B.V., Veldhuis, M., Maatman, A. & de Baar, H. Physiological responses of three species of marine pico-phytoplankton to ammonium, phosphate, iron and light limitation. *J. Sea Res.* **53**, 109-120 (2005).
23. Davidson, K. & Gurney, W. An investigation of non-steady-state algal growth. II. Mathematical modelling of co-nutrient-limited algal growth. *J. Plankt. Res.* **21**, 839-858 (1999).
24. Fasham, M., Flynn, K., Pondaven, P., Anderson, T. & Boyd, P. Development of a robust marine ecosystem model to predict the role of iron in biogeochemical cycles: A comparison of results for iron-replete and iron-limited areas, and the SOIREE iron-enrichment experiment. *Deep Sea Res. Part I* **53**, 333–366 (2006).
25. Calbet, A. *et al.* Impact of micro-grazers and nano-grazers on phytoplankton assessed by standard and size-fractionated dilution grazing experiments. *Aquat. Microb. Ecol.* **50**, 145-156 (2008).
26. Broglio, E., Saiz, S., Calbet, A., Trepat, I., & Alcaraz, M. Trophic impact and prey selection by crustacean zooplankton on the microbial communities of an oligotrophic area (NW Mediterranean Sea). *Aquat. Microb. Ecol.* **35**, 65-78 (2004).
27. Lacroix, G. & Grégoire, M. Revisited ecosystem model (MODECOGeL) of the Ligurian Sea: seasonal and interannual variability due to atmospheric forcing. *J. Mar. Sys.* **37**, 229-258 (2002).
28. Anderson, T.R. & Pondaven, P. Non-Redfield carbon and nitrogen cycling in the Sargasso Sea: pelagic imbalances and export flux. *Deep Sea Res. Part I* **50**, 573-591 (2003).
29. Thingstad, T., Skjoldal, E. & Bohne, R. Phosphorus cycling and algal–bacterial competition in Sandsfjord, western Norway. *Mar. Ecol. Progr. Ser.* **99**, 239-259 (1993).
30. Vrede, K., Heldal, M., Norland, S. & Bratbak, G. Elemental composition (C,N,P) and cell volume of exponentially growing and nutrient-limited bacterioplankton. *App. Environ. Microbiol.* **68**, 2965-2971 (2002).
31. Ward, B., Dutkiewicz, S., Jahn, O. & Follows, M.J. A size-structured food-web model for the global ocean. *Limnol. and Oceanogr.* **57(6)**, 1877-1891 (2012).
